# IGFBP2 Plays an Essential Role in Cognitive Development during Early Life

**DOI:** 10.1002/advs.201901152

**Published:** 2019-10-14

**Authors:** Shumsuzzaman Khan, Xinjiang Lu, Qingyao Huang, Jiawei Tang, Jian Weng, Zhi Yang, Minchao Lv, Xiaokang Xu, Fangyuan Xia, Mengchen Zhang, Yi Li, Shuangshuang Liu, Gareth Leng, Nicholas Spitzer, Jizeng Du, Xuequn Chen

**Affiliations:** ^1^ Institute of Neuroscience Department of Neurobiology, and Department of Neurology of Second Affiliated Hospital NHC and CAMS Key Laboratory of Medical Neurobiology Key Laboratory of Medical Neurobiology of Zhejiang Province Zhejiang University School of Medicine Hangzhou 310058 China; ^2^ Experimental Physiology University of Edinburgh Edinburgh EH8 9XD UK; ^3^ University of California, San Diego La Jolla CA 92093‐0357 USA; ^4^ Present address: Case Western Reserve University Cleveland Ohio USA

**Keywords:** hippocampus, IGFBP2, IGFR1, learning and memory, neuropeptides

## Abstract

Identifying the mechanisms underlying cognitive development in early life is a critical objective. The expression of insulin‐like growth factor binding protein 2 (IGFBP2) in the hippocampus increases during neonatal development and is associated with learning and memory, but a causal connection has not been established. Here, it is reported that neurons and astrocytes expressing IGFBP2 are distributed throughout the hippocampus. IGFBP2 enhances excitatory inputs onto CA1 pyramidal neurons, facilitating intrinsic excitability and spike transmission, and regulates plasticity at excitatory synapses in a cell‐type specific manner. It facilitates long‐term potentiation (LTP) by enhancing N‐methyl‐d‐aspartate (NMDA) receptor‐dependent excitatory postsynaptic current (EPSC), and enhances neurite proliferation and elongation. Knockout of *igfbp2* reduces the numbers of pyramidal cells and interneurons, impairs LTP and cognitive performance, and reduces tonic excitation of pyramidal neurons that are all rescued by IGFBP2. The results provide insight into the requirement for IGFBP2 in cognition in early life.

## Graphical abstract

Insulin‐like growth factor binding protein 2 (IGFBP2) regulates plasticity in a cell‐type‐specific manner at excitatory synapses in the hippocampus, facilitates long‐term potentiation (LTP) by IGFR1 and N‐methyl‐d‐aspartate receptor (NMDAR)‐s/evoked excitatory postsynaptic currents (eEPSCs), and rescues LTP and cognitive performance and excitation in KO mice.

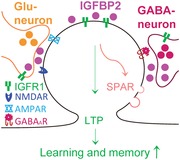

## 1 Introduction

Understanding the mechanisms of early cognitive development has great importance as it could help to improve cognitive therapy.^1^, ^2^, ^3^ The IGF family includes neurotrophic factors and mediates learning and memory.^1^, ^2^, ^3^ Sensory experience regulates cortical inhibition by IGF1^3^ and IGF1 restores synaptic deficits.^4^ IGF‐1 receptors regulate transmission via mitochondrial mediation in the hippocampus.^5^ IGF‐1 also restores corticospinal axon‐dependent functions in adult mice and is a growth facilitator required for axon regeneration in adult mice and rats with spinal cord injury.^6^, ^7^ IGF‐II enhances memory and prevents forgetting via IGF‐II receptors.^1^

The expression of insulin‐like growth factor binding protein‐2 (IGFBP2) in the hippocampus increases during postnatal development and is associated with learning and memory, but how it affects cognitive enhancement is unknown. IGFBP2 is one of six members of the superfamily of IGF‐binding proteins, most of which are synthesized in the liver and released into the circulation to regulate the bioavailability of IGFs I and II and their tissue distribution.^2^, ^8^ IGFBP2 is present in the central nervous system from the embryo and neonate to adulthood and is highly expressed in the developing brain, secreted by both primary astrocytes and fetal neurons. IGFBP2 expression is correlated with brain development, astrocyte proliferation, and neurite outgrowth. Its expression is coordinated with that of IGF1 in the cerebellum and in developing sensory networks, but not in the hippocampus,^2^, ^9^ suggesting that it may have a hippocampus‐specific function.

Here we investigate whether IGFBP2 is essential for cognitive development and how it affects information processing in the hippocampus. Using transgenic mice deficient in IGFBP2 (*igfbp2^−/−^*) we show that IGFBP2 is necessary for spine growth and neuronal proliferation in postnatal mice, and that it acts as a neuromodulator and coordinator of Hebbian and homeostatic plasticity in a cell‐type specific manner. Through these actions it integrates metaplastic signals to facilitate long‐term potentiation (LTP) and spatial learning and memory.

## 2 Results

### 2.1 IGFBP2 Is Required for Normal Hippocampal Development

**Figure 1:**
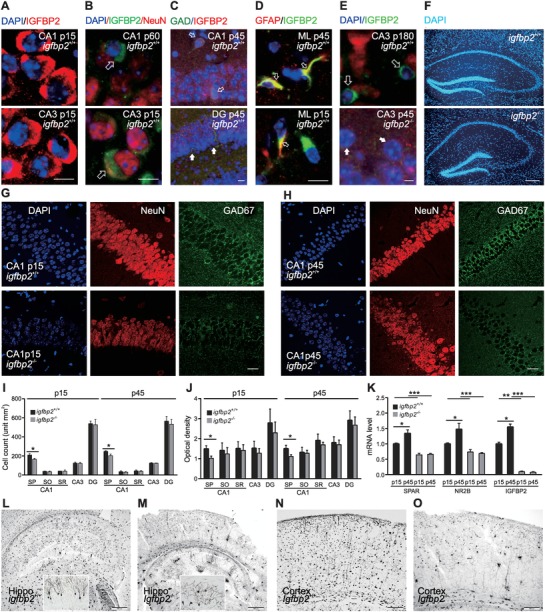
Distribution of IGFBP2‐positive cells and total cell counts and morphology in the hippocampus of wild‐type and mutant mice. A–E) IGFBP2 neurons and astrocytes in the hippocampus of wild‐type mice (*igfbp2* ^+/+^). Arrows in B, neurons expressing IGFBP2 and NeuN; open arrows in C, small neurons expressing GAD67 and IGFBP2; solid arrows, larger neurons expressing GAD67 without IGFBP2, D) Astrocytes expressing IGFBP2, E) Neurons expressing IGFBP2 in wild‐type and not in *igfbp2* ^−/−^ mice. F) DAPI staining in the hippocampus of wild‐type and *igfbp2* ^−/−^ mice. G,H) Staining with DAPI, NeuN, and GAD67 in CA1 from wild‐type and *igfbp2^−/−^* mice at p15 and p45. I,J) Quantification of total cell number and optical density in the stratum oriens (SO), stratum pyramidale (SP), and stratum radiatum (SR) of CA1, CA3, and the DG (*n* = 4 mice). K) Expression of IGFBP2, NR2B, and SPAR. L–O) Golgi‐stained neurons in cortex and hippocampus. K) *P < 0.05, **P < 0.01, ***P < 0.001, two‐tailed t‐tests and Bonferroni's multiple comparison. Data are mean ± SEM. Scale bar, 20 µm in A–E, G, H; 200 µm in F, N, O; 500 µm in L, M (50 µm, inset).

IGFBP2‐immunopositive neurons were distributed throughout the hippocampus (CA1, CA3, and DG) and in cortical layers 1 and 2/3 in wild‐type *igfbp2^+/+^* mice at postnatal days 15, 45, and 60 (p15, p45, and p60) (**Figure** **1**A–D and Figure S1A, Supporting Information). In the hippocampus, IGFBP2 was expressed in pyramidal neurons (Figure 1A,B) and in GABAergic interneurons (Figure 1C), while IGFBP2‐positive astrocytes were found in the molecular layer (Figure 1D). IGFBP2 was expressed in GABAergic interneurons in CA1, while in the DG there were GABAergic interneurons (glutamic acid decarboxylase (GAD)‐positive cells) without IGFBP2 immunostaining in *igfbp2^+/+^* mice (Figure 1C). At p180, IGFBP2 neurons were fewer in number and restricted to CA3, while, as expected, *igfbp2^−/−^* p45 mice failed to show IGFBP2‐immunoreactivity (Figure 1E).

There was no marked difference in the size and shape of the brain and hippocampus between wild‐type and *igfbp2^−/−^* mice (Figure 1F–H), but *igfbp2^−/−^* mice had fewer cells and a lower optical density in the stratum pyramidale (SP) of the CA1 region than wild‐type mice (Figure 1G–J and Figure S1A–E, Supporting Information), and markedly fewer interneurons throughout the hippocampus (CA1, CA3, and the DG) and cortex (layers 1 and 2/3) (Figure S1C,D, Supporting Information). There was also lower expression of transcripts of the N‐methyl‐d‐aspartate receptor (NMDAR) subunit NR2B and spine‐associated‐Rap‐specific GTPase‐activating protein (SPAR) in the hippocampus and cortex (Figure 1K and Figure S1F, Supporting Information) in *igfbp2^−/−^* mice at postnatal day (p)15 and p45. Meanwhile, in the cortex and hippocampus of wild‐type mice, Golgi staining revealed that neuronal dendrites were more richly branched than in *igfbp2^−/−^* mice (Figure 1L–O). Thus, IGFBP2 is important for hippocampal development.

### 2.2 IGFBP2 Enhances Excitatory Synaptic Transmission

**Figure 2:**
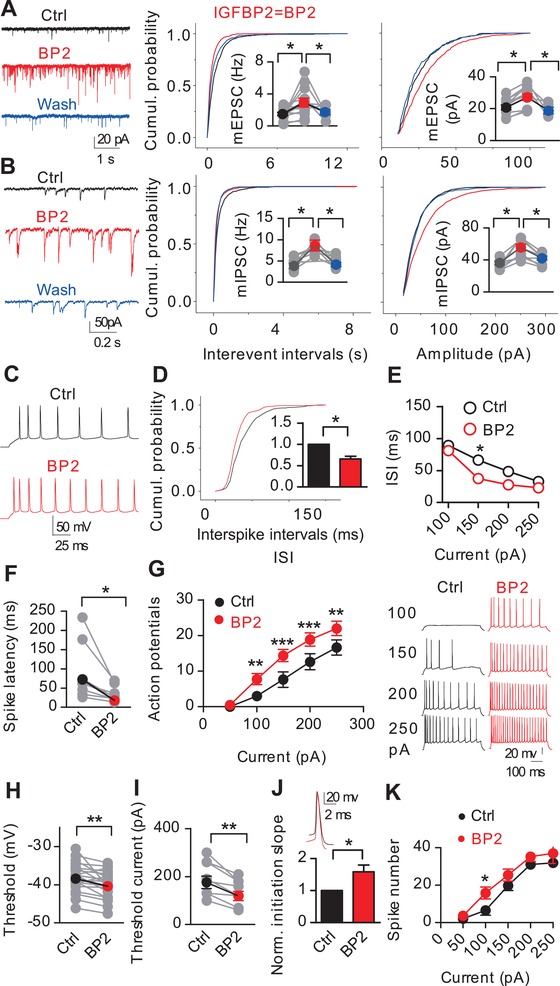
Excitatory and inhibitory responses and excitability of p14‐17 CA1‐pyramidal neurons to IGFBP2. A,B) Exemplar mEPSCs/mIPSCs in pyramidal neurons at –70 mV and the cumulative distributions of their frequency and amplitude (DNQX, AP5, and TTX added in incubation. *n* = 11 EPSCs, 12 IPSCs, 4‐6 washouts. C–G) Exemplar spikes, cumulative probability, plots of interspike interval, spike latency, and pyramidal neuron excitability (*n* = 8). H–J) Voltage threshold (*n* = 19), threshold current (*n* = 8), and normalized slope of initiation for spikes (*n* = 8). K) CA1‐FSI excitability (*n* = 5). Two‐sample Kolmogorov‐Smirnov test in panels A, B, and D; otherwise, paired two‐tailed t‐tests. * *P* < 0.05, ** *P* < 0.01, ***P < 0.001; data are mean ± SEM.

Electrical activity is a characteristic of neuronal development, neural circuit maturation, and activity‐dependent cognition, particularly in the postnatal period.^10^ To investigate whether IGFBP2 can alter the efficacy of synaptic transmission, we maintained slices of mouse hippocampus (p14‐17) in vitro and exposed them to IGFBP2 while recording the electrical activity of pyramidal neurons in the CA1 region. IGFBP2 increased the frequency and amplitude of both miniature excitatory postsynaptic currents (mEPSCs) and miniature inhibitory postsynaptic currents (mIPSCs) (**Figure** **2**A,B), indicating enhanced release of both excitatory and inhibitory neurotransmitters.

IGFBP2 induced a significant increase spike activity of pyramidal neurons with a decreased interspike interval when depolarized with a series of 500 ms step currents. The latency of evoked spikes was reduced (Figure 2C–G) through a reduction in spike threshold and an increased normalized slope of initiation (Figure 2H–J). During IGFBP2‐enhanced spiking, there was no change in the amplitude or half‐width of spikes, in the post‐spike after hyperpolarization, or in the resting membrane potential (RMP) (Figure S2A, Supporting Information).

This IGFBP2‐enhanced excitability could be due to either the activation of excitatory inputs or the suppression of inhibitory inputs. Dividing the increase in firing rate (11.8 spikes/s/neuron, Figure 2G) by the increase in mEPSC frequency (2.2 Hz per neuron, Figure 2A) revealed that each pyramidal neuron received ≈5.3 excitatory inputs for each spike. However, IGFBP2 had no significant effect on the firing rate of fast‐spiking interneurons in CA1 (FSIs) or regular‐spiking interneurons (RSIs) (Figure 2K and Figure S2B–D, Supporting Information), and had no significant effect on their voltage threshold (Figure S2E,F, Supporting Information). Thus, IGFBP2 activates excitatory synapses on pyramidal neurons without affecting inhibitory inputs from FSIs and RSIs. Treatment with an IGFR1 antagonist (IGFR1A) prevented this enhancement (Figure S2G,H, Supporting Information). Treatment with IGFR1A alone reduced the frequency and amplitude of sEPSCs (Figure S3I, Supporting Information), suggesting that endogenous IGFBP2 regulates excitatory transmission to pyramidal neurons via IGFR1.

**Figure 3:**
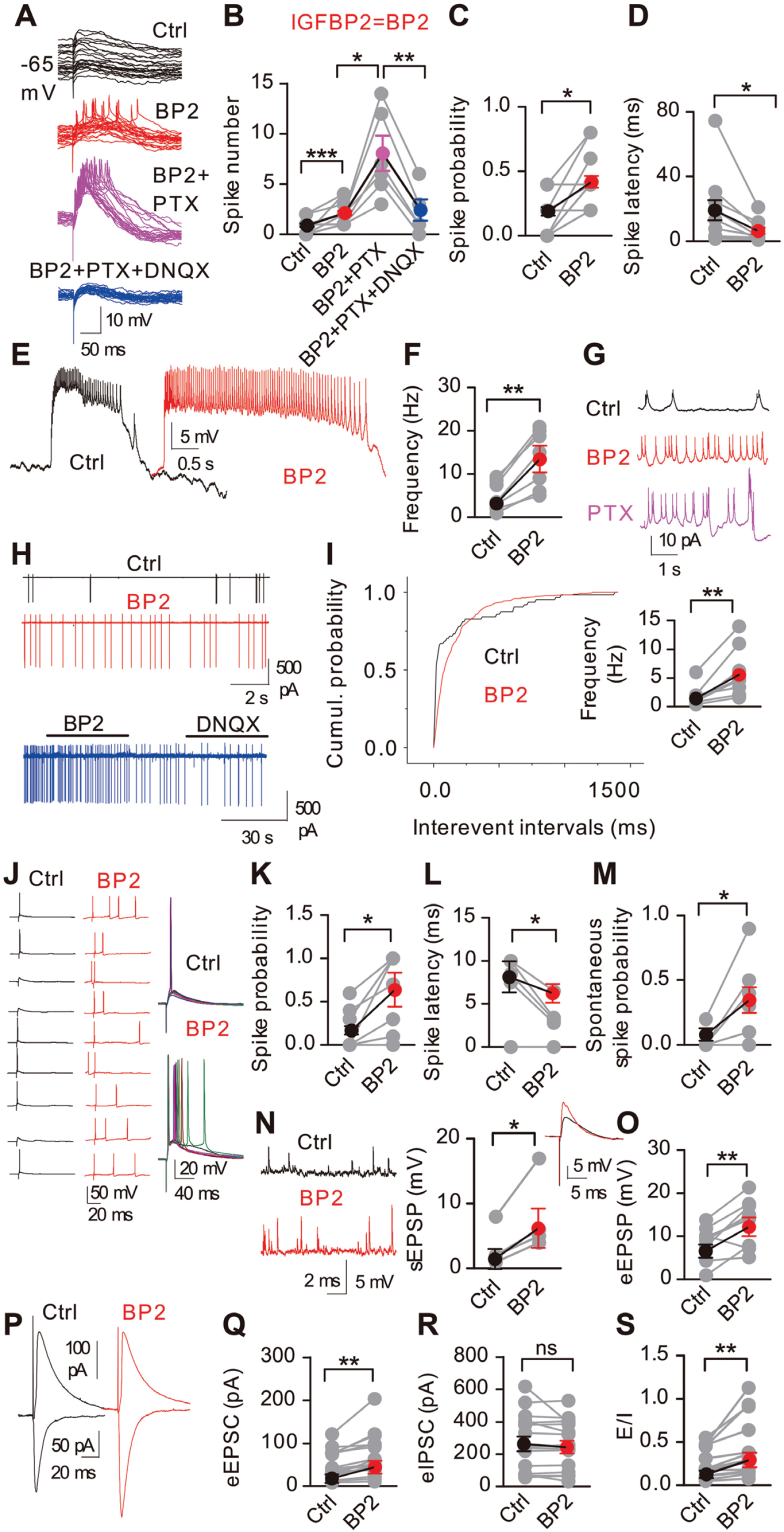
IGFBP2 enhances the fidelity of spike transmission. A) Exemplar cell‐attached spikes (CA1 pyramidal neurons) evoked by SC stimulation. B–D) Spike number, spike probability (*n* = 19), and spike latency (*n* = 11) after IGFBP2 treatment. E–I) Exemplar cell‐attached spontaneous spiking under current‐clamp (*n* = 7) and voltage‐clamp (*n* = 8). J–M) Exemplar whole‐cell spikes evoked by SC stimulation with spike probability (*n* = 9), spike latency (*n* = 6), and spontaneous spike probability (*n* = 5). N,O) Exemplar traces and group data of s/eEPSPs (*n* = 4, 9) by SC stimulation. P–S) Exemplar eIPSCs and eEPSCs evoked by SC stimulation. IGFBP2 enhanced eEPSCs without affecting eIPSCs, and increased the E/I ratio (*n* = 15). Two‐sample Kolmogorov‐Smirnov test in panel O; paired two‐tailed t‐tests elsewhere. *P < 0.05, **P < 0.01; data are mean ± SEM.

To investigate whether IGFBP2 affects the fidelity of spike transmission, we made cell‐attached and whole‐cell current‐clamp recordings from pyramidal neurons and evoked spikes synaptically by stimulating Schaffer collaterals (SCs). IGFBP2 significantly increased the number of spikes evoked by SC stimulation while decreasing the spike latency (**Figure** **3**A–D), accompanied by enhanced spontaneous spiking (Figure 3E–I). Similar results were found in whole‐cell recordings for evoked (Figure 3J–L) and spontaneous spiking (Figure 3M), and were accompanied by enhanced spontaneous and evoked EPSPs (Figure 3N,O). Thus IGFBP2‐evoked excitation reflects enhanced EPSP‐spike coupling. In the presence of IGFBP2, the γ‐aminobutyric acid (GABA) receptor antagonist picrotoxin (PTX) enhanced both spontaneous and evoked spiking of pyramidal neurons that were abolished by the glutamate receptor antagonist 7‐dinitroquinoxaline‐2,3‐dione (DNQX). Thus, IGFBP2‐activation occurred in the continued presence of GABAergic inhibition, either from “bilingual” pyramidal neurons that use both GABA and glutamate as neurotransmitters^11^ or from interneurons.

Since neuronal firing results from the balance between synaptic excitation and inhibition, we investigated the effect of IGFBP2 on this balance. We found that IGFBP2 enhanced the evoked eEPSCs but not the eIPSCs (Figure 3P–S), and then investigated how IGFBP2 enhanced the eEPSCs. IGFBP2 had no significant effect on the paired‐pulse ratios (PPRs) of either EPSCs or IPSCs (Figure S3A,B, Supporting Information), suggesting that it did not affect presynaptic function. Alternatively, IGFBP2 may have increased the number of functional synapses. One way of measuring the relationship between EPSCs and the underlying quantal synaptic responses is with the coefficient of variation (CV) of EPSC amplitude, since 1/CV^2^ is correlated with the probability of release. We found an increase in 1/CV^2^ for eEPSCs with IGFBP2 and a concomitant decrease in 1/CV^2^ for eIPSCs (Figure S3C, Supporting Information).

In the presence of IGFBP2, the 1/CV^2^ of eEPSCs was positively correlated with both the eEPSC amplitude and excitation/inhibition (E/I) ratio, while the 1/CV^2^ of eIPSCs showed no such correlation (Figure S3D–G, Supporting Information). No correlation was found between the PPR of EPSCs and the eEPSC amplitude either in the presence or absence of IGFBP2, while the negative correlation between the PPR of IPSCs and the eIPSC amplitude was weaker in the presence of IGFBP2 (from *p* = 0.01 to *p* = 0.04) (Figure S3H,I, Supporting Information), suggesting that IGFBP2 might be involved in use‐dependent depression of IPSCs.^12^ Since GABAergic synapses express bidirectional plasticity in the neonatal hippocampus,^13^, ^14^ it appears that IGFBP2 may increase functional excitatory synapses with a concomitant decrease or no reduction of GABAergic synapses (Figure S3J, Supporting Information).

To learn how the interaction between EPSCs and IPSCs determines the final synaptic signal, we configured whole‐cell voltage‐clamp recording at −40 mV for EPSCs, 0 mV for IPSCs, or −80 mV to record both currents from the same cells. IGFBP2 significantly increased both the frequency and amplitude of sEPSCs (confirmed by blocking with DNQX) and postsynaptic currents (PSCs; the sum of EPSCs and IPSCs), but not those of sIPSCs (Figure S4A–C, Supporting Information). In the presence of IGFBP2, the PSC frequencies became correlated with EPSC but not IPSC frequencies (Figure S4D,E, Supporting Information). We obtained the same results for spontaneous amplitudes (Figure S4F,G, Supporting Information). Thus, in the presence of IGFBP2, increased EPSCs enhance the excitation–inhibition (E/I) ratio (Figure S4H,I, Supporting Information).

### 2.3 IGFBP2 Increases in the E/I Ratio Are Cell‐Type Specific

**Figure 4:**
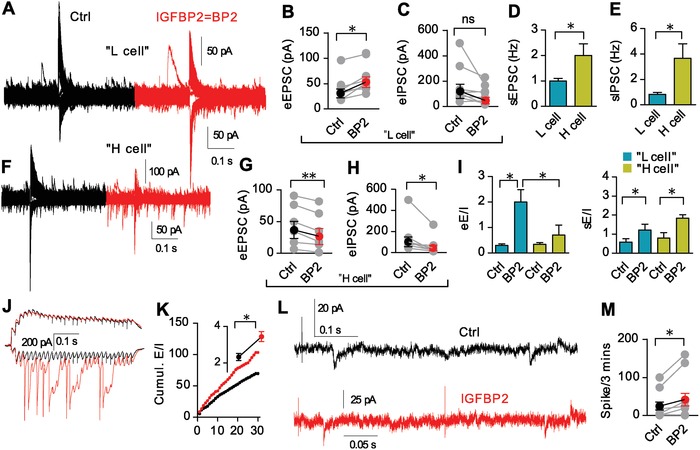
IGFBP2 coordinates L and H cells plasticity in a cell‐type specific manner. A–C) Exemplar recordings from CA1 pyramidal L cells with eEPSC and eIPSC amplitude (*n* = 8). D,E) Comparison of spontaneous activity in L and H cells. F–H) Exemplar recordings from CA1 pyramidal H cells with eEPSCs and eIPSCs (*n* = 6). I) Comparison of eE/I and sE/I in L and H cells (*n* = 6). J,K) Exemplar IPSCs (upper) and EPSCs (lower) evoked by 60 Hz stimulation and cumulative E/I ratios (*n* = 10). L) Exemplar simultaneous recording of sEPSCs, sIPSCs, and action potentials. M) IGFBP2 enhanced excitation (*n* = 7). Paired two‐tailed t‐test except panels D and E. *P < 0.05, **P < 0.01; data are mean ± SEM.

To gain an overview of synaptic potentiation, we recorded evoked as well as spontaneous activity from the same cells. We found that there are two different responses to IGFBP2 in electrophysiologically different pyramidal neurons in CA1. In pyramidal neurons that displayed lower frequencies of either sEPSCs or sIPSCs [≈1 Hz; termed “L‐cells”], IGFBP2 enhanced the eEPSC but had no significant effect on the eIPSCs (**Figure** **4**A–C, Figure S5A,B, Supporting Information). By contrast, in cells with higher spontaneous activity [≥2±0.5 Hz; termed “H‐cells”]; Figure 4D–F and Figure S5C, Supporting Information), IGFBP2 reduced both the eEPSCs and the eIPSCs (Figure 4G,H). Thus IGFBP2 has cell‐type specific effects and L cells are the principal target of the elevated E/I ratio (Figure 4I). Neither IGFBP2 nor high‐frequency stimulation (HFS) significantly increased the sIPSC amplitude in either L or H cells (Figure S5B,C, Supporting Information), while suppression of the eIPSCs in H cells suggests potentiation and stabilization of excitatory synapses.

We next asked how the plasticity of L and H cells is affected by IGFBP2. In the presence of IGFBP2, we found a positive correlation between sEPSC frequency and eEPSC amplitude in L cells (Figure S5D, Supporting Information) but not in H cells (Figure S5E, Supporting Information). However, there was no such correlation for inhibitory synaptic activity (sIPSC vs eIPSC) in either L or H cells in either the presence or knockout of IGFBP2 (Figure S5F,G, Supporting Information). A positive correlation between eEPSC and eIPSC amplitudes in both L and H cells (Figure S5H,I, Supporting Information) indicates that the E/I ratios are similar in CA1 pyramidal neurons of both types, while the absence of such a correlation in the presence of IGFBP2 in L cells (Figure S5H, Supporting Information) and a negative correlation in H cells (Figure S5I, Supporting Information), suggests that a shift in the E/I ratio is cell‐type specific and IGFBP2 dependent.

During 60 Hz stimulation of the SCs, IGFBP2 increased the eEPSC amplitude but not the eIPSC amplitude, resulting in a cumulatively enhanced eE/I ratio (Figure 4J,K and Figure S5J, Supporting Information). Notably, the stimulus trains evoked a nearly homogeneous pattern of EPSC amplitudes (Figure 4J), indicating that activity is unsaturated during development. This was confirmed by E–I dynamics with spontaneous firing (Figure 4L,M).

### 2.4 Interruption of E‐I Balance in *igfbp2^−/−^* Mice

**Figure 5:**
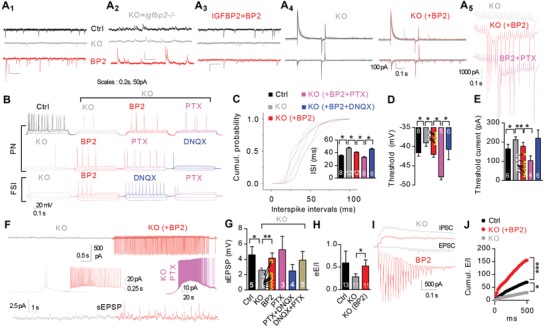
IGFBP2 maintains the E–I balance in *igfbp2^−/−^* mice. A) Exemplar CA1 pyramidal neuron A_1_) sEPSCs, A_2_) sIPSCs, A_3_) PSCs, A_4_) eEPSCs–eIPSCs, and A_5_) EPSCs evoked by 60 Hz stimulation. Slices from p14‐17 mice. B) Exemplar spikes from pyramidal neurons and FSIs in *igfbp2^+/+^* (Ctrl) and *igfbp2^−/−^* (KO) mice. C) Cumulative probability for interspike intervals (ms) among different groups. D,E) Changes in the voltage threshold and threshold current (*n* = 12). F,G) Exemplar spikes (upper: whole‐cell; middle: cell‐attached) and cell‐attached sEPSPs (*n* = 12). H) The E/I ratio was restored after IGFB2 treatment. I,J) Exemplar EPSCs and IPSCs evoked by 60 Hz stimulation and cumulative E/I ratios (*n* = 18). Paired two‐tailed t‐test in panels D, E, G, H, J. * *P* < 0.05, ** *P* < 0.01; data are mean ± SEM.

The frequencies of sEPSCs and sIPSCs were lower in *igfbp2^−/−^* mice than in wild‐type mice with no differences in amplitude (**Figure** **5**A_1‐2_, Figure S6A, Supporting Information). Pyramidal neurons showed prolonged interspike intervals, a more depolarized voltage threshold, and an increased threshold current, resulting in decreased spiking and a prolonged spike latency (Figure 5B–E and Figure S6B,C, Supporting Information). In addition, decreased spontaneous firing was found in cell‐attached and whole‐cell recordings in both voltage‐ and current‐clamp modes (Figure 5F and Figure S6D, Supporting Information) and sEPSP amplitudes were decreased (Figure 5G). Exogenous IGFBP2 (500 × 10^−9^ m)^15^ rescued these changes as well as the excitability of the pyramidal neurons (Figure S7A–C, Supporting Information), suggesting that it plays a crucial role in synaptic homeostasis and excitability. There were no significant differences in spike shape, resting membrane potential or membrane input resistance (*R*_In_) between control and *igfbp2* ^−/−^ mice (Figure S6E, Supporting Information).

The correlation between excitatory and inhibitory inputs onto pyramidal neurons was interrupted in *igfbp2* ^−/−^ mice and rescued after IGFBP2 treatment for 20 min (Figure S7A,B, Supporting Information). Normalized EPSC‐ and PSC‐frequencies and amplitudes in neurons from either wild‐type or *igfbp2* ^−/−^ mice were positively correlated after IGFBP2, while IPSC and PSC frequencies and amplitudes were positively correlated in wild‐type but not in *igfbp2* ^−/−^ mice and were less strongly correlated in wild‐type mice after IGFBP2 treatment (Figures S4D,F and S7C,D, Supporting Information). Neither sEPSCs nor sIPSCs were correlated with spiking in *igfbp2* ^−/−^ mice. After IGFBP2 treatment only sEPSCs but not sIPSCs were correlated with spiking (Figure S7E,F, Supporting Information), suggesting that increased excitatory inputs control the spiking of CA1 pyramidal neurons in the presence of IGFBP2. The reduced eE/eI and sE/sI‐frequency but not the sE/I‐ amplitude was rescued after IGFBP2 treatment in *igfbp2* ^−/−^ neurons due to enhanced excitation (Figure 5A_4_,H and Figure S7G–J, Supporting Information).

In response to high‐frequency (60 Hz) stimulation, pyramidal neurons in *igfbp2* ^−/−^ mice showed a shallower train of EPSC amplitudes (Figure 5I) than in wild‐type mice (Figure 5J) that was rescued by IGFBP2 (Figure S7K,L, Supporting Information) with no effect on cumulative IPSC amplitudes (Figure S7M,N, Supporting Information). Thus IGFBP2 restored the E/I ratios (Figure 5J and Figure S7O, Supporting Information). The last few EPSCs of the train were larger than the first few (Figure 5I), while IPSCs showed the converse pattern. After IGFBP2 treatment we found two types of rescued patterns of EPSC amplitudes: either the first few EPSCs in the train were smaller (Figure 5A_5_) or the first few EPSCs were larger (Figure 5I), further suggesting that IGFBP2 regulates synaptic function in a cell‐type‐specific manner.

### 2.5 IGFBP2 Regulates LTP and Water Maze Learning and Memory

**Figure 6:**
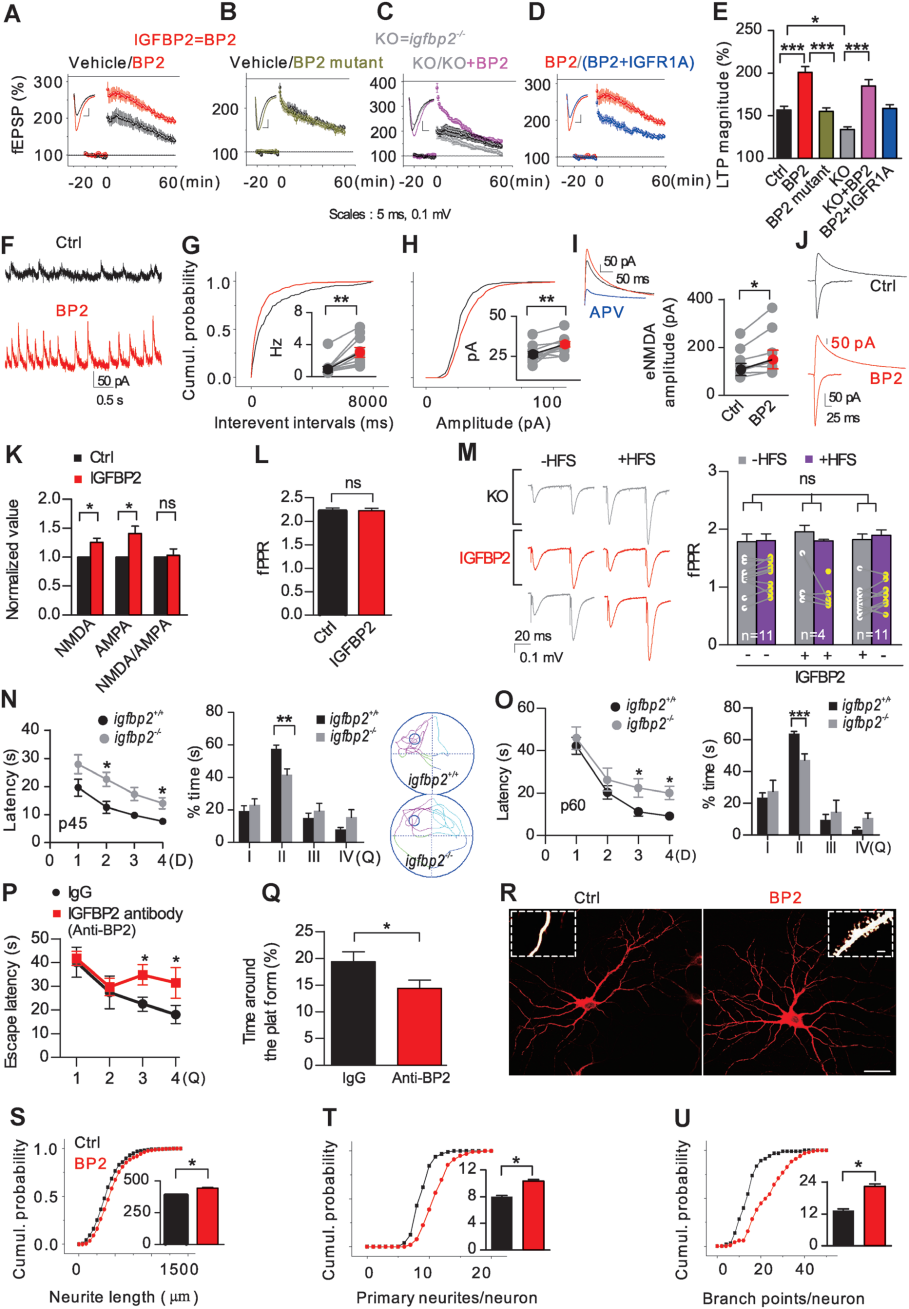
Mechanism of IGFBP2‐mediated LTP enhancement. A,B) IGFBP2 facilitated LTP (*n* = 10) but mutant IGFBP2 did not (*n* = 8). C) LTP in *igfbp2^−/−^* slices was rescued by IGFBP2 treatment (*n* = 10). D) IGFBP2 failed to maintain stable LTP in IGFR1A‐treated *igfbp2^+/+^* slices (*n* = 6). E) Comparison of LTP magnitudes (ANOVA). F–I) Exemplar s/eNMDAR currents in CA1 pyramidal neurons at +40 mV from SC stimulation, and cumulative distribution of frequency G and spontaneous H (*n* = 11), or evoked amplitudes I (*n* = 7). J) Exemplar eNMDAR and eAMPAR currents. K,L) Exemplar traces of field EPSPs evoked at 50 ms intervals and field PPR (*n* = 11) after IGFBP2 treatment in *igfbp2^+/+^* and *igfbp2^−/−^*slices. M) Normalized NMDA (*n* = 7) and AMPA (*n* = 13) currents and NMDA/AMPA ratios. N,O) Escape latency (*F*_(1,72)_ = 11.15, *P* < 0.05) and time spent in each quadrant (p45 and p60), and representative movement traces at p45 (*n* = 10). P,Q) Longer escape latency in the training phase and less time around the platform in probe tests in mice injected with IGFBP2 antibody (control, IgG = 7; IGFBP2 antibody = 8); and absence of significant differences between IgG and IGFBP2 antibody‐injected mice in the latency of visual tests and swimming speed (data not shown). R) Representative cultured hippocampal neurons and dendritic spines (insets). S–U) Neurite length, and numbers of primary neurites and branch points, after IGFBP2 treatment (*n* = 105). One‐way ANOVA with Bonferroni's correction in panels O and P, two‐tailed t‐test. *P < 0.05, **P < 0.01, ***P < 0.001; data are mean ± SEM. Scale bars, 20 µm in R (5 µm, inset).

We then asked how IGFBP2 affects Hebbian plasticity in L cells in slices from neonatal wild‐type mice. In the presence of IGFBP2, HFS elicited stronger LTP in CA1 pyramidal neurons (200.9 ± 3.9%) than in controls (vehicle, 156.7±4.2%), while treatment with mutant‐IGFBP2^15^ had no effect (155.2 ± 3.9%) (**Figure** **6**A,B). In slices from *igfbp2* ^−/−^ mice, HFS induced a transient synaptic potentiation (133.9 ± 3.1%), while IGFBP2 elicited a large initial potentiation with poorly maintained LTP magnitude (185.1 ± 7.4%, Figure 6C). IGFR1A suppressed the IGFBP2‐induced LTP (158.8 ± 4.3%, Figure 6D,E). IGFBP2 induced no LTP at GABAergic synapses (data not shown). IGFBP2 significantly increased the frequency and amplitude of spontaneous NMDA‐mediated EPSCs with a concomitant increase in NMDA‐mediated EPSCs evoked by SC stimulation that was blocked by the NMDA receptor antagonist APV (25 × 10^−6^ m) (Figure 6F–I). Since IGFBP2 increased both NMDA and AMPA (α‐amino‐3‐hydroxy‐5‐methyl‐4‐isoxazolepropionic acid) currents (Figure 6J), NMDA/AMPA ratios were preserved (Figure 6K) following activity‐dependent synaptic scaling. No significant differences were found in the field PPR in CA1 pyramidal neurons between wild‐type and *igfbp2^−/−^* mice before and after HFS, either in the presence or absence of IGFBP2 (Figure 6L,M).

When we assessed hippocampus‐dependent learning and memory in the Morris water maze at p45 and p60, *igfbp2* ^−/−^ mice showed a slower learning profile (*F*_(1,72)_ = 11.15, *P* < 0.05) than wild‐type mice. In memory‐retention tests, they also spent less time exploring the former location of the platform (Figure 6N,O). A similarly poor performance with a decreased PSD‐95 was seen in wild‐type mice microinjected with IGFBP2 antibody through cannulae implanted bilaterally in the hippocampus (Figure 6P,Q and Figure S8A, Supporting Information). Similar escape latencies and swimming speeds during training and retention trials in control IgG and IGFBP2 antibody‐treated mice revealed that the impaired performance of the latter mice was not due to deficits in vision or motor activity (data not shown). These results indicate that IGFBP2 is specifically involved in learning and memory.

Examination of the impact on synaptic morphology in cultured hippocampal neurons showed that IGFBP2 (500 × 10^−9^ m) induced an increase in total neurite length and numbers of primary dendrites and secondary branch points per neuron after 3 d (*n* = 105, Figure 6R–U). The early induction of dendritic spines in ≈5% of neurons (Figure 6R, inset) suggests that IGFBP2 increases the number of synapses. No changes in hippocampal IGF1 and IGF2 levels were seen in response to microinjection of IGFBP2 in WT and KO mice (Figure S8B,C, Supporting Information). Interestingly, corticotrophin‐releasing factor (CRF, 0.1 × 10^−9^ m), increased IGFBP2 release via CRFR1 receptors in cultured hippocampal neurons and *igfbp2^−/−^* mice had lower levels of CRF mRNA in the hippocampus (Figure S9A,B, Supporting Information). Intermittent hypoxia (16% O_2_) enhanced IGFBP2, IGFR1, CRF, and CRFR1 expression in the neonatal hippocampus (Figure S9C–E, Supporting Information). IGFR1 was enriched in dendritic spines/filopodia and boutons and colocalized with CRFR1 in cultured hippocampal neurons (Figure S9F, Supporting Information). At 12.5 × 10^−9^ m (but not 50 × 10^−9^ or 25 × 10^−9^ m), CRF‐induced robust LTP (183.8 ± 8.9%) was suppressed by IGFR1A (143.3 ± 3.6%), to a level similar to that of the effect on LTP amplitude in the presence of IGFR1A alone (148.5 ± 4.2%) (Figure S9G–L, Supporting Information). The response to a CRFR1 antagonist was similar in the presence of IGFBP2 (127.3 ± 6.6%). Thus there is cross‐talk between CRFR1 and IGFR1.

## 3 Discussion

The IGFBP2‐knockout (KO) (IGFBP2^−/−^) and wild‐type (IGFBP2^+/+^) mice in our study were healthy and fertile and had similar body weights at birth and p45–p60; furthermore, HE staining of the hippocampus showed no significant difference between the KO and wild‐type groups, consistent with previous findings.^16^ However, the Morris water maze performance of wild‐type mice was better than for KO mice at p45 and p60.

Our study confirms IGFBP2 expression by pyramidal neurons, GABAergic interneurons, and astrocytes in the hippocampus and cortex of the developing mouse brain.^17^ The distribution of IGFBP2‐positive cells in the hippocampus suggests that IGFBP2 acts there by an autocrine and/or paracrine mechanism to influence synaptic activity and learning and memory. As neonates grew, the number of IGFBP2‐positive neurons increased as did the mRNA levels of IGFBP2, SPAR, and NR2B; hippocampal IGFBP2 protein increased gradually from p14 to p35, along with PSD‐95 and SPAR proteins. SPAR localizes in dendritic spines and forms a complex with PSD‐95 and NMDAR.^18^ In contrast, *igfbp2* ^−/−^ mice had diminished levels of SPAR mRNA and NR2B mRNA and reduced numbers of hippocampal cells and interneurons, indicating involvement of IGFBP2 in NMDAR‐dependent learning and memory via alteration of dendritic spines as reported for IGF‐II.^1^ IGFBP2 is also involved in brain disorders as well as cognitive functions. The numbers of IGFBP2‐positive cells decreased in the hippocampus of a mouse model of amyotrophic lateral sclerosis, and serum IGFBP2 levels were lowered in patients with bipolar disease and depression.^2^ IGFBP2 plays a key role in neurite outgrowth and the early induction of spines, as well as SPAR cytoskeletal construction and NR2B expression. Thus, IGFBP2 is synthesized and released in the hippocampus and is important for the development of axons and dendritic spines^19^, ^20^ and in learning and memory.

IGFBP2 is required for E‐I balance in a cell‐type‐specific manner. We found that IGFBP2 enhanced both the evoked and spontaneous neuronal excitability and the fidelity of spike transmission of CA1 pyramidal neurons by ≈5.3 excitatory inputs. CA1 pyramidal neurons containing IGFBP2 may also enable parallel information processing^21^ in cell‐type‐specific firing patterns^22^ since we found IGFBP2 did not interfere with CA1‐interneuron spiking. IGFBP2‐positive neurons are a subpopulation in the hippocampus and IGFBP2 may be a new neuromodulator at the hippocampal microcircuit level.

Synaptic strength results from the integration of excitatory and inhibitory synaptic inputs. Here we demonstrated that IGFBP2 enhanced both mEPSCs and mIPSCs, revealing bidirectional homeostatic scaling. On the other hand, IGFBP2 selectively enhanced sEPSCs while sparing sIPSCs in the presence of action potentials. Taken together, on a competitive basis IGFBP2 showed a selective bias toward excitatory rather than inhibitory input by simultaneous upregulation of evoked and spontaneous excitatory synaptic input via an overlapping or independent mechanism.^23^ Interestingly, *igfbp2^−/−^* mice manifested not only a drop in sEPSC frequency but also in sIPSCs and this was rescued after IGFBP2 treatment, consistent with the idea that neurons maintain the E‐I balance by autonomously decreasing their inhibitory inputs in response to excitatory synaptic dysfunction.^24^ We found that IGFBP2 also enhanced evoked synaptic excitation in pyramidal neurons of the hippocampus. The excitation of pyramidal neurons was diminished in *igfbp2* ^−/−^ mice and was rescued by application of IGFBP2 for 20 min, indicating that IGFBP2 can stabilize nascent synapses.^25^ Binding of IGFBP2 to IGFR1 induces a transient increase in intracellular calcium concentration^5^ that may be responsible for synaptic stability. Thus, IGFBP2, as a member of a growth factor family, performs a significant regulatory role in synapses.

Using electrophysiological techniques, we identified a previously unrecognized pyramidal cell‐type in the CA1 region with distinct neurophysiological properties in response to IGFBP2. Among the pyramidal neurons, IGFBP2 preferentially excited L cells rather than H cells. Corelease of glutamate and GABA in the presence of IGFBP2 may be responsible for shunting inhibitory currents, facilitating excitation by cell‐type‐specific orchestration of the electrical properties of pyramidal neurons. This process can contribute to the activity‐dependent modulation of synaptic connectivity that is important for hippocampal development. IGFBP2 may assist in the redistribution of excitatory and inhibitory activity between the two types of pyramidal neurons during spatial learning.^21^, ^22^

IGFBP2 enhanced mEPSC frequency without changing the PPR, indicating a novel role of IGFBP2 in glutamate receptor kinetics.^26^, ^27^ Since inhibitory synapses can be unstable during development,^13^ enhancement of mIPSCs by IGFBP2 can allow neurons to stabilize activity without changing the relative strength of synaptic inputs. Studies of *igfbp2^−/−^* mice revealed that IGFBP2 regulated the E‐I balance during early life. We noted a ≈50% reduction of E/I‐ratios in *igfbp2* ^−/−^ compared to controls that was rescued after IGFBP2 treatment, indicating that IGFBP2 enhances information transmission and E–I dynamics. Despite the complexity of the distribution of E/I ratios, these data are consistent with the notion that activity remains unsaturated during development. Furthermore, FSIs inhibit somatostatin‐expressing interneurons to regulate the output of pyramidal neurons,^28^ so excitation of FSIs by IGFBP2 may alter interneuronal–pyramidal neuron connections. Thus IGFBP2 enhanced excitatory transmission and increased the E‐I balance in a cell‐type specific manner in the hippocampus.

IGFBP2 regulates LTP via IGFR1 signaling and crosstalk with CRFR. IGFBP2 increased the number of functional synapses and enhanced LTP by activating both spontaneous and evoked AMPAR and NMDAR currents via an IGFR1 signaling pathway. Impaired LTP and poor performance in the water maze by *igfbp2^−/−^* mice confirmed the involvement of IGFBP2 in spatial learning and memory.

Our results support an emerging role of IGFBP2 as a key associative signal with the stress hormone CRF driving activity‐dependent plasticity to flexibly tune neural circuits. The relationship between stress and synaptic plasticity takes the form of an inverted‐U, and the hippocampus is necessary for the enhancement and impairment of learning after stress.^29^ Meanwhile, hippocampal IGFBP2 and IGFR1 protein increased gradually from p28 to p35 in mice and was enhanced further after the mild postnatal stress of intermittent hypoxia. Persistent increases in SPAR expression as well as LTP were reported in mice exposed to intermittent hypoxia in a previous study from our lab. Deletion of insulin receptor and IGFR1 in mice hippocampus decreased expression of glutamate receptor 1 protein in synaptosomes, impaired cognition, and increased anxiety behaviors.^30^ Together, these findings suggest that there is cross‐talk between stressors and learning and memory during early life. Experiments on a rat model of post‐traumatic stress have also proposed that IGFBP2 has a therapeutic‐like antidepression effect.^31^ Microarray analysis showed markedly increased‐IGFBP2 mRNA in α‐secretase cleaved amyloid precursor protein (a neuroprotective)‐treated organotypic hippocampus slice culture.^32^ Consistently, using the hippocampal transcriptome and RT‐PCR, robust activation of hippocampal IGFBP2 in a middle‐aged female rat model of menopause with estradiol replacement has shown that *Igfbp2* contributes to neurogenesis, neuroplasticity, and memory.^33^ Thus, IGFBP2 is a potential target for learning and memory impairment therapies and also for the treatment of neurodegenerative diseases.

## 4 Conclusions

In summary, we found that IGFBP2 enhances excitatory inputs onto CA1 pyramidal neurons, facilitating their intrinsic excitability and spike transmission, and regulates plasticity at excitatory synapses in a cell‐type specific manner. It contributes to LTP facilitation by enhancing spontaneous and evoked NMDAR excitatory postsynaptic currents by enhancing neurite proliferation and elongation. Knockout of *igfbp2* leads to reduced numbers of pyramidal cells and interneurons, which impairs LTP and cognitive performance and reduces the tonic excitation of pyramidal neurons, all of which are rescued by addition of IGFBP2. Our results provide a novel insight into the role of IGFBP2 in cognition during early life.

## 5 Experimental Section

*Animals*: Wild‐type C57BL/6J mice (certification no. SCXK2008‐0033) were purchased from the Experimental Animal Centre of Zhejiang Province (China) and group housed under a 12/12‐h light/dark cycle (lights on 06:00‐18:00) at 20 ± 2 °C with free access to food and water for one week prior to experiments. All animal procedures complied with the National Institutes of Health guidelines using protocols approved by the Institutional Animal Care and Use Committee of Zhejiang University (ZJU201304‐1‐01‐025). Mutant *igfbp2^−/−^* mice on the C57BL/6J genomic background (MGI: 96437) were purchased from UC Davis (B6;129S5‐*igfbp2* ^Gt(OST365171)Lex^/Mmucd, mmrrc:011721‐UCD) and F2 and F3 generations were used according to institutional guidelines. Genotyping was performed by PCR with genomic DNA extracted from tail tips. Primers used for *igfbp2* ^−/−^ genotyping were: for mutant forward: 5′‐GGGTTCTCCTGGCTGGTGACTC‐3′; reverse: 5′‐ATAAACCCTCTTGCAGTTGCATC‐3′; and wild‐type forward: 5′‐GGGTTCTCCTGGCTGGTGACTC‐3′; reverse: 5′‐GAGTCTCCCTGGATCTGA TTAAGG‐3′.

*Electrophysiology*: Male wild‐type C57BL/6J mice at p14‐17 were anesthetized by isoflurane inhalation or with 90 mg kg^−1^ sodium pentobarbital before decapitation. The brain was extracted and the left hippocampus was dissected out and glued to the platform of a semiautomatic vibrating‐blade microtome (VT1000; Leica). The platform was then placed in a slicing chamber containing artificial cerebrospinal fluid (aCSF) at 4 °C. Hippocampal slices (350 µm) were prepared in ice‐cold aCSF consisting of (in × 10^−3^ m) 125 NaCl, 3 KCl, 1.25 NaH_2_PO_4_, 2 MgSO_4_, 2 CaCl_2_, 25 NaHCO_3_, 15 glucose and 0.4 l‐ascorbic acid. All slices were incubated in a custom‐made interface holding chamber submerged in aCSF at 34 °C for 1 h and then maintained at 27 °C for up to 8 h until they were transferred to the recording chamber. Slices were superfused with aCSF at 29–31 °C (2 mL min^−1^ for whole‐cell and 3 mL min^−1^ for extracellular recording). All solutions were saturated with 95% O_2_ and 5% CO_2_.

*Whole‐Cell Recording*: Slices were visualized through an infrared‐sensitive CCD camera with a 40 × water‐immersion lens (Olympus) and recorded using whole‐cell techniques (MultiClamp 700B Amplifier, Digidata 1440A analog‐to‐digital converter) and pClamp 10.2 software (Axon Instruments/Molecular Devices). Slices were allowed 10–30 min to equilibrate before recording. Recording electrodes were pulled on a horizontal puller (Sutter P‐1000). For whole‐cell recordings, the access resistance was monitored by a hyperpolarizing step of –5 mV every 60 s; experiments were discarded if the series resistance exceeded 20%. Miniature EPSCs and sEPSCs were recorded from pyramidal neurons in voltage‐clamp using glass pipettes with a tip resistance of 2–4 MΩ and an internal solution containing (in × 10^−3^ m) 130 K gluconate, 20 KCl, 10 HEPES buffer, 4 Mg‐ATP, 0.3 Na‐GTP, 10 disodium phosphocreatine, and 0.2 EGTA, pH 7.3 with KOH, and 288 mOsm. Gabazine (10 × 10^−6^ m), DNQX (10 × 10^−6^ m), TTX (100 × 10^−9^ m), and IGFBP2 (1 × 10^−6^ m) were delivered in the bathing solution during recordings. For mIPSC recording, we used a high Cl^−^ internal solution containing (in × 10^−3^ m) 130 CsCl, 4 NaCl, 10 TEA‐Cl, 10 HEPES, 0.2 EGTA, 10 Tris‐phosphocreatine, 4 Mg‐ATP and 0.5 Na‐GTP, pH 7.3 with CsOH, and 288 mOsm. Recordings of sEPSCs, sIPSCs, PSCs from the same neuron were based on reversal potentials as described previously.^12^ The holding potential for sEPSC = −40 mV, sIPSC = 0 mV, and PSC = −80 mV. No antagonists were added to the bath in these experiments, except for EPSCs that were confirmed by DNQX exposure at the end of recordings. Spontaneous frequency and peak amplitude were measured with the Mini Analysis program (Synaptosoft).^34^

*Action Potential Recording*: For whole‐cell‐evoked action potential recording either by current injection or SC stimulation, glass pipettes (2–4 MΩ) were filled with a solution containing (in × 10^−3^ m) 130 K gluconate, 20 KCl, 10 HEPES buffer, 4 Mg‐ATP, 0.3 Na‐GTP, 10 disodium phosphocreatine and 0.2 EGTA, pH 7.2 with KOH, and 288 mOsm. The inter‐spike intervals (ISI), calculated for averaged intervals between sequential action potentials in a train elicited in response to a 500 ms suprathreshold current of 50 to 250 pA, were used to quantify the excitability of CA1 pyramidal neurons and FSIs. Shape parameters were measured from action potentials evoked by 500 ms current injection. The action potential threshold was measured as the minimal membrane potential value at the time corresponding to the peak of the third derivative of the membrane potential, as described previously,^34^ using custom routines written in the Mini Analysis program (Synaptosoft). Offline analysis was performed using custom routines written in Clampfit 10.2, Prism 5, and Origin8 pro.

*Same‐Cell eEPSCs, eIPSCs, and PPR Recording*: During same‐cell recording of eEPSCs, eIPSCs, and PPR, the same pipette solution was used as for m/sEPSC recording, in which K gluconate and KCl were replaced by CsMeSO_3_ and CsCl, respectively. eEPSCs and eIPSCs were recorded at their respective reversal potentials for EPSCs (0/+10 and −60 mV).^35^ The PPR was determined to estimate release probability. The peak amplitudes were evoked by two identical electrical stimuli separated by 50 ms and the PPR was calculated as the ratio of the peak amplitudes of EPSC‐IPSC_2_/EPSC‐IPSC_1_. 60‐84 individual traces were recorded to measure the average PPR.

*Same‐Cell Recording of Action Potentials, s/eEPSCs, s/eIPSCs and PSCs*: K^+^‐based pipette solution was used for EPSC, IPSC and action potential recordings and the holding potential was gradually changed from −60 to 0/+10 mv. EPSC/IPSC traces were evoked by 60 Hz stimulation (30 stimuli). Four individual traces were averaged to measure cumulative EPSC/IPSC amplitudes. After action potential recording in the whole‐cell current‐clamp mode, the MultiClamp 700B Amplifier was switched to the voltage‐clamp mode and EPSCs/IPSCs were recorded after a minimum of 2 min. In the case of cell‐attached recording of action potentials, the time between current‐ and voltage‐clamp recordings was at least 1 min, so that recovery from short‐term modification was complete.

*Same‐Cell Simultaneous Recording of Spontaneous EPSCs, IPSCs, and Action Potentials*: K^+^‐based pipette solution was used for same‐cell simultaneous recording of sEPSCs, sIPSCs, and action potentials in voltage‐clamp mode, with a holding potential at which the amplifier current, *I*_amp_ = 0 pA.

*NMDAR‐EPSCs Recording*: For s/eNMDAR current recording, the Cs^+^‐based pipette solution was used. s/eNMDAR EPSCs were recorded at a holding potential of +40 mV in the presence of PTX and DNQX. NMDAR currents were evoked using a tungsten bipolar electrode placed in the SC excitatory afferents from area CA3 to deliver stimuli.

*EPSPs*: Whole‐cell spontaneous and SC‐stimulation‐evoked EPSPs were recorded from CA1 pyramidal neurons using the K^+^‐based pipette solution in current‐clamp mode. For cell‐attached sEPSP recording the pipettes were filled with modified aCSF (1.5 × 10^−3^ m KCl).

*Cell‐Attached Recording*: For cell‐attached measurement of spontaneous and SC‐evoked action potentials, results were pooled from recordings in modified aCSF (1.5 × 10^−3^ m KCl for spontaneous firing; 1 × 10^−3^ m CaCl_2_ and 1 × 10^−3^ m MgSO_4_ for evoked spiking).

*Extracellular Recording*: Field EPSPs were evoked at 0.05 Hz as previously described^36^ and recorded with an aCSF‐filled pipette (1–2 MΩ) positioned in the stratum radiatum of CA1; SC inputs were stimulated with monophasic pulses using a bipolar concentric electrode placed in CA3. LTP was induced by HFS consisting of four 1 s trains of 100 Hz pulses, delivered 20 s apart or four trains delivered 5 min apart, with the stimulus intensity set at 20–30% of the spike threshold. Field potentials were analyzed using Origin8 software.

*Cell Culture*: Hippocampal neurons were cultured as described previously. Briefly, embryonic hippocampal neurons (E18) were dissociated in Ca^2+^‐ and Mg^2+^‐free Hank's balanced salt solution containing 0.125% tyrosine and triturated in Dulbecco's modified Eagle's medium (DMEM)/10% fetal bovine serum (FBS), and plated on poly‐d‐lysine‐coated 60 mm dishes at 1 000 000 cells per well. Cultures were grown in serum‐free medium for 21 d before being used for biochemical experiments and the medium was changed every 5 d. Dendritic morphology was studied in cultures of 2500 cells per coverslip grown for only 5 d and for 14–18 d. IGFBP2 (500 × 10^−9^ m, 3 d) was directly applied to cultured neurons and saline was added as control.

*Immunocytochemistry and Neurite Morphology Analysis*: Cultured hippocampal neurons at 3 d in vitro were treated with IGFBP2 and grown for an additional 5 d for neurite analysis. Cells were fixed for 10 min in ice‐cold 4% paraformaldehyde, permeabilized with 0.2% Triton‐X‐100 in phosphate‐buffered saline (PBS), and blocked with 5% BSA (bovine serum albumin) for 1 h. Subsequently, cells were incubated with primary rabbit antibody to MAP2 (1:1,000, Millipore) in 5% BSA for 3 h at room temperature. Coverslips were washed three times with washing buffer (0.05% Triton‐X‐100 in PBS) for 10 min each, incubated with secondary antibodies conjugated with Alexa Fluor 488 and 555 (1:1000, Invitrogen, Carlsbad, CA) in 5% BSA for 1 h at room temperature, and washed three times with washing buffer for 10 min each. Coverslips were mounted in Fluoroshield mounting medium containing 4, 6‐diamidino‐2‐phenylindole (DAPI) (Sigma). Images were acquired with a 60× oil‐immersion objective (NA 1.4) on an inverted confocal microscope (Olympus FV1000) by investigators blind to the experimental condition. Three parameters of neurite growth were analyzed using MetaMorph image analysis software, after capturing images of MAP2‐positive neurons with cell body diameters of 15‐20 µm: total length of neurite (µm), number of primary neurites and number of branch points per cell. Application settings were adjusted at the beginning of analysis and kept the same for all images in the experiment. Images of 15–20 neurons per condition were captured in six independent experiments. The following primary antibodies were used: anti‐IGFR1 antibody (1:500, Milliopre‐MABS192)/rabbit anti‐IGFR1 (1:200, Sigma) and anti‐CRFR1 (1:200, R&D SYSTEM‐MAB3930); colocalization was determined using FV10‐ASW 4.1 Viewer software.

*Immunohistochemistry*: Mice were anesthetized by intraperitoneal injection of ketamine (100 mg kg^−1^) and xylazine (10 mg kg^−1^) and transcardially perfused with PBS at pH 7.4 followed by 4% paraformaldehyde in PBS. Brains were removed and further fixed overnight in 4% paraformaldehyde. An HM 450 Sliding Microtome (Thermo Scientific) was used to cut coronal sections at 20 µm. Sections were blocked with 1% BSA, 2% normal goat serum (NGS), and 0.3% TritonX‐100 in PBS at room temperature for 1 h and incubated with primary antibodies in working buffer (0.1% BSA, 0.2% NGS, and 0.3% TritonX‐100 in PBS) at 4 °C overnight. The following primary antibodies were used: goat anti‐IGFBP2 (1:200, Santa Cruz), mouse anti‐NeuN (1:1000; Millipore), mouse anti‐GAD67 (1:500, Millipore), and anti‐GFAP (1:500, Abcam). Sections were washed four times with working buffer for 10 min each, incubated with secondary antibodies conjugated with Alexa Fluor 488, 594, or 633‐647 (1:500 or 1:1000, Life Technologies) in working buffer for 1 h at room temperature, and then washed four times with working buffer for 10 min each. Sections were mounted in Vectashield mounting medium containing DAPI (1:1000 in PBS, 25 min). Images were captured on a Nikon A1R scanning laser microscope or an Olympus FV1000/olyVIA microscope using a 60×/100× oil immersion objective and processed using MetaMorph software and NIH ImageJ. Immunofluorescence images were converted to black‐and‐white and cells were counted with MetaMorph by experimenters blind to the test conditions. The total cells and interneurons in the hippocampus (SP, SO, SR in CA1, CA3, and the DG) and cortex (L1 and L2/3) of *igfbp2* ^+/+^ and *igfbp2* ^−/−^ mice (p15 and p45) were stained for DAPI and GAD67 respectively. Regions of interest (mm^2^) of CA1, CA3, DG, L1, and L2/3) were selected and the number of cells and interneurons were counted using MetaMorph image‐analysis software. Four sections (20 µm, bilateral or left hippocampus and cortex) from each mouse (*n* = 4) were counted and the data are presented as mean ± SEM.

*Golgi Staining*: Brain tissue was rinsed in impregnation mixing Solutions A and B at room temperature for 2 weeks and transferred to Solution C for 72 h to 1 week. Sections were cut at 30–40 µm; each section was checked under the microscope with Solution C and then rinsed in Solutions D and E mixed 10 times (Rapid GolgiStain Kit, PK 401/401A, FD Neurotechnologies, Inc., Columbia, MD).

*Morris Water Maze*: Morris water maze tests were performed using adult (p45/p60) male control (C57BL/6J) wild‐type and *igfbp2* ^−/−^ mice. Mice were handled for 5 min each for three consecutive days before beginning experiments. The maze consisted of a large circular tank (1 m in diameter, 0.5 m high) of water at 25 ± 1 °C made opaque by the addition of nontoxic water‐based white paint. An escape platform (11 cm in diameter) was submerged 0.5–1 cm below the water surface in the center of one of the four quadrants and remained in this position for each mouse. Several visual cues were placed on the walls of the behavioral room as spatial references. An automated tracking system (DigBehv‐MWM; Jiliang Software Technology Co. Ltd., Shanghai, China) monitored performance using the following parameters: escape latency (finding the submerged platform), swimming speed, and a visual sensitivity test.

*Real Time Quantitative RT‐PCR*: Total RNA was prepared using TRIzol reagent (Life Technologies). RNA (2 µL) was reverse‐transcribed with the TransScript TM RT enzyme mix and then stored at −20 °C. First‐strand cDNA was subjected to PCR amplification using a Quantitect SYBR Green PCR kit (Qiagen, Valencia, CA). The primer sequences were as follows: IGFBP2 (forward: 5′‐CTTCCTTCTGGCGTTGGGAG‐3′; reverse: 5′‐TTCATGCCTGACTTGAGGGG‐3′), IGFR1 (forward: 5′‐GACTTCGGACCAGTCTCGC‐3′; reverse: 5′‐GAGGAGCAAAGCCCAAATCG‐3′), CRF (forward: 5′‐AAAATGTGGATCCAAGGAGGA‐3′; reverse:5′‐TAGCCACCCCTCAAGAATGAA‐3′), CRFR1 (forward:5′‐CACTACCATGTTGCAGTCATC‐3′; reverse: 5′‐CGAACATCCAGAAGAAGTTGG‐3′), CRFR2 (forward: 5′‐TACCGAATCGCCCTCATTGT‐3′; 5′‐CCACGCGATGTTTCTCAGAAT‐3′), SPAR (forward: 5′‐GGCAGAGAAGTGAGGACAG‐3′; reverse: 5′‐ATGGCCTTGCTTGTTTGGAG‐3′), NR2B (forward: 5′‐GGTCTTTGCTTCTACGGGCT‐3′; reverse: 5′‐GTGAGCCAGAGAGCTTCCAG‐3′), 18S RNA (forward: 5′‐GTAACCCGTTGAACCCCATT‐3′; reverse: 5′‐CCATCCAATCGGTAGTAGCG‐3′). Quantitative real‐time PCR was carried out on a 7500 Real‐Time PCR System.

*Western Blotting*: Hippocampal extracts were obtained by Polytron homogenization in cold lysis buffer containing protease and phosphatase inhibitors (0.2 m NaCl, 0.1 m HEPES, 10% glycerol, 2 × 10^−3^ m NaF, 2 × 10^−3^ m Na_4_P_2_O_7_, 4 U mL^−1^ aprotonin, 2 × 10^−3^ m DTT, 1 × 10^−3^ m EGTA, 1 × 10^−6^ m microcystin, and 1 × 10^−3^ m benzamidine). Protein concentrations were determined using the BioRad protein assay (BioRad Laboratories, Hercules, CA). Equal amounts of total protein (10–20 µg per lane) were resolved on denaturing SDS‐PAGE gels and transferred to Hybond‐P membranes (Millipore, Billerica, MA) by electroblotting. Membranes were dried, reactivated in methanol for 5 min, and washed with three changes of water. Membranes were then blocked in 3% milk/PBS or according to manufacturers' instructions for 1 h at room temperature, and incubated with either anti‐IGFBP2 (1:200, Santa Cruz Biotechnology or Abcam, ab136494), or anti‐actin (1:5000, Santa Cruz Biotechnology) antisera in PBS overnight at 4 °C. Anti‐SPAR antibody and Anti‐SIPA1 were from Abcam (Ab1925436) and anti‐IGFR1 antibody from Sigma. Colloidal gold total protein stain was from BioRad. Membranes were washed, treated with a secondary HRP‐labeled donkey anti‐rabbit antibody (1:4000, GE Healthcare, Waukesha, WI) for 1 h, washed again, and incubated with HRP‐streptavidin complex and ECL detection reagents (GE healthcare, Waukesha, WI).

*ELISA*: IGFBP2, IGF1, IGF2, and CRF levels in cultured neurons and hippocampal tissue supernatants were quantified using ELISA as previously described, according to the product protocol (IGFBP2 ELISA kit: BOSTER, catalog number EK0385; CRF ELISA kit: Peninsula Laboratories International, Inc., catalog number S‐1181.0001; IGF1 ELISA kit: Abcam, Ab100695; IGF2 ELISA kit:SEA051Mu, Cloud‐clone Corp).

*Implant Surgery and Stereotactic Microinjection*: Hippocampal microinjection was performed as previously described. p35 mice were implanted with bilateral cannulae aimed at the dorsal hippocampus. Animals were anesthetized by sodium pentobarbital (80 µg g^−1^, i.p.) and mounted in a Kopf stereotaxic frame that was used to position the 24‐gauge stainless steel guide cannulae in the dorsal hippocampi (AP −1.8 mm, ML ± 1.5 mm, DV −1.3 mm). Coordinates were chosen based on a mouse brain atlas. Mice were housed individually and allowed at least 7 d of postoperative recovery before being used in behavioral experiments. At p42, anti‐IGFBP2 antibody (Abcam, ab136494) or vehicle (PBS, pH 7.4) was infused into the dorsal hippocampus, then the water maze test was performed and hippocampal tissue was removed for Western blotting. At p60, peptide fragment IGFBP2^15^ (500 × 10^−9^ m) was infused into dorsal hippocampus in WT and KO mice for ELISA and Western blotting.

For infusions, the injectors were inserted into the guide cannulae and left in place for 1 min followed by a 1 min infusion of antibody (1.5 nmol in 0.5 µL per side, dissolved in 500 nL PBS) or vehicle (500 nL of PBS). The injectors were left in place for 1 min after the infusion and then replaced with obturators. To verify proper placement of the cannulae, mice were anesthetized and perfused with 4% paraformaldehyde in PBS at the end of the behavioral experiments. Their brains were postfixed overnight in the same fixative with 30% sucrose. Coronal sections (40 µm) were cut through the hippocampus, stained with cresyl violet, and examined under a light microscope. Mice with incorrect cannula placements were discarded from the study.

*Hypoxia Exposure*: After birth, newborn male mice and their mothers were immediately exposed to hypoxia simulating an altitude of 2000 m (16.0% O_2_ at sea level) in a well‐ventilated hypobaric chamber (FLYDWC50‐IIC; Avic Guizhou Fenglei Aviation Armament Co., Ltd.). Control newborn mice were set at sea level (21.0% O_2_) in a similar chamber. Intermittent hypoxia was applied from 08:00 to 12:00 daily (4 h per day) as previously described.^37^ Mice at p9, p14, p28, and p35 were deeply anesthetized with ether and decapitated and the hippocampi were removed, immediately frozen in liquid nitrogen, and stored at −80 °C.

*Drugs and Reagents*: All drugs and buffers for intracellular and extracellular solutions, as well as ATP, GTP, phosphocreatine, DNQX, AP5, and picrotoxin were from Sigma. The synthetic peptide containing the heparin‐binding domain (amino‐acids 196‐199) and nine additional amino‐acids of mouse IGFBP2, ^188^KHLSLEEPKKLRP^200^ (referred to as HBD peptide),^15^ and control peptide for HBD (AALSLEEPAALAP), called mutant IGFBP2, were from China Peptide, dissolved in ddH_2_O and stored at −20 °C until use within 6 months of purchase. JB‐1 was from Bachem. Gabazine, CRF, and TTX were from Tocris Bioscience. CP‐154,526 was kindly donated^38^ by the Pfizer Company (Pfizer Inc., Groton, CT, USA).

*Statistical Analysis*: All data are shown as mean ± SEM. unless otherwise noted. *P* < 0.05 was considered to be statistically significant. Data for which a specific *P* value is not indicated are not significantly different (*P < 0.05, **P < 0.01, ***P < 0.001). For electrophysiology experiments, significance was determined by the paired two‐tailed *t*‐test for direct comparisons between neurons, the Kolmogorov‐Smirnov test for frequency and sometimes for amplitude, the two‐tailed *t*‐test for comparisons between populations, and one‐way ANOVA with Bonferroni post‐hoc for LTP. To assess correlations, the best‐fit linear regression is shown as indicated in the figure legends. Behavioral assays were analyzed by one/two‐way ANOVA with the Bonferroni post‐hoc test. For imaging, qRT‐PCR, ELISA, and western blot experiments, significance was determined by the two‐tailed *t*‐test.

## Conflict of Interest

The authors declare no conflict of interest.

## Supporting information

Supplementary
