# Supplementary material for: IGFBP2 Plays an Essential Role in Cognitive Development during Early Life
Source: Adv Sci (Weinh). 2019 Oct 14;6(23):1901152. doi: 10.1002/advs.201901152 (PMC6891907; doi:10.1002/advs.201901152)
Supplement: Supplementary file 1 — Supplementary [file ADVS-6-1901152-s001.pdf]

Copyright WILEY-VCH Verlag GmbH & Co. KGaA, 69469 Weinheim, Germany, 2019.

# Supporting Information

for *Adv. Sci.,* DOI: 10.1002/advs.201901152

# IGFBP2 Plays an Essential Role in Cognitive Development during Early Life

*Shumsuzzaman Khan, Xinjiang Lu, Qingyao Huang, Jiawei Tang, Jian Weng, Zhi Yang, Minchao Lv, Xiaokang Xu, Fangyuan Xia, Mengchen Zhang, Yi Li, Shuangshuang Liu, Gareth Leng, Nicholas Spitzer, Jizeng Du, and Xuequn Chen**

Copyright WILEY-VCH Verlag GmbH & Co. KGaA, 69469 Weinheim, Germany, 2019.

# Supporting Information

**IGFBP2 Plays an Essential Role in Cognitive Development during Early Life**

*Shumsuzzaman Khan, Xinjiang Lu, Qingyao Huang, Jiawei Tang, Jian Weng, Yang Zhi, Minchao Lv, Xiaokang Xu, Fangyuan Xia, Mengchen Zhang, Yi Li, Shuangshuang Liu, Gareth Leng, Nicholas Spitzer, Jizeng Du, and Xuequn Chen**

S. Khan, X. Lu, Q. Huang, J. Tang, J. Weng, Z. Yang , M. Lv, Y. Li, S. Liu, X. Xu, F. Xia, M. Zhang, Prof. J Du, Prof. X. Chen

Institute of Neuroscience, and Department of Neurology of Second Affiliated Hospital, NHC and CAMS Key Laboratory of Medical Neurobiology, Key Laboratory of Medical Neurobiology of Zhejiang Province, Zhejiang University School of Medicine, Hangzhou 310058, China

E-mail: chewyg@zju.edu.cn

Prof. G. Leng

Experimental Physiology, University of Edinburgh, Edinburgh EH8 9XD, UK

Prof. N. Spitzer

University of California, San Diego, La Jolla, CA 92093-0357, USA

**Figure S1-9 and Toc Figure**
**Experimental Section**

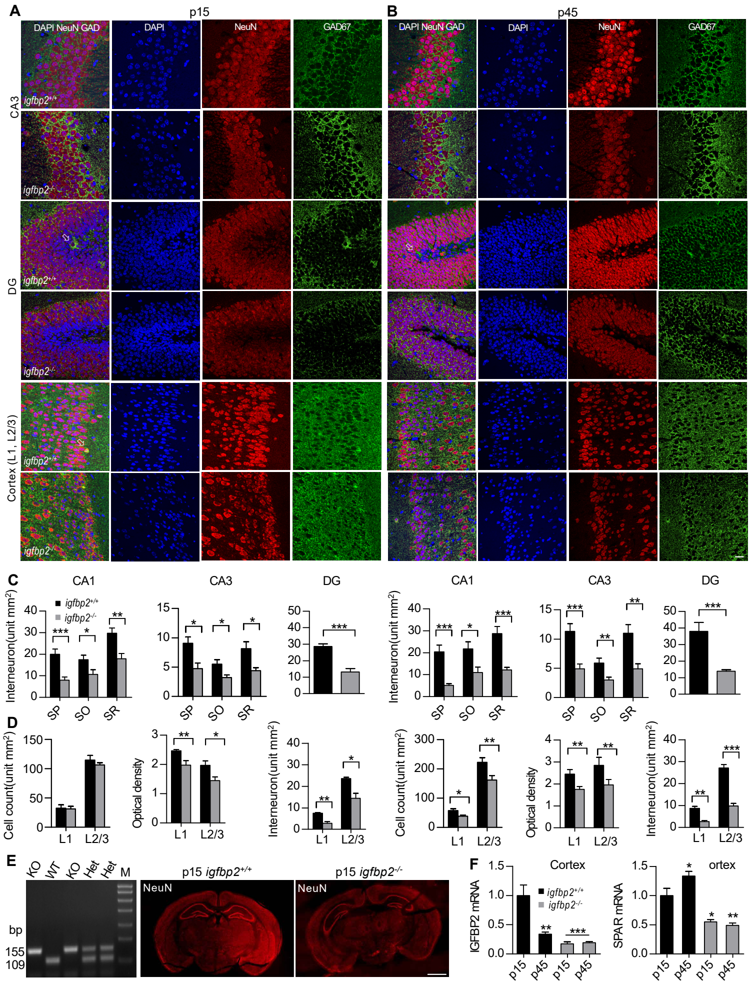


**Figure S1.** Number and density of hippocampal and cortical interneurons. A,B) DAPI, NeuN, and GAD67 staining of CA3, DG, L1, and L2/3 from wild-type (n = 6) and *igfbp2*$^{-/-}$ mice (n = 4) at p15 and p45. Arrows indicate interneurons in the DG. C) Counts of interneurons from hippocampus (CA1, CA3, and DG) per unit area (mm$^2$). D) Cell counts and density of interneurons in cortex (per mm$^2$). E) Genotyping and NeuN staining of brain sections. F) Whole cortical IGFBP2 and SPAR mRNA normalized to p15 (n = 6). * $P$ <0.05, ** $P$ <0.01, *** $P$ <0.001, two-tailed t-test; data are mean ± SEM. Scale bars, 20 μm in A, B; 1 mm in E.

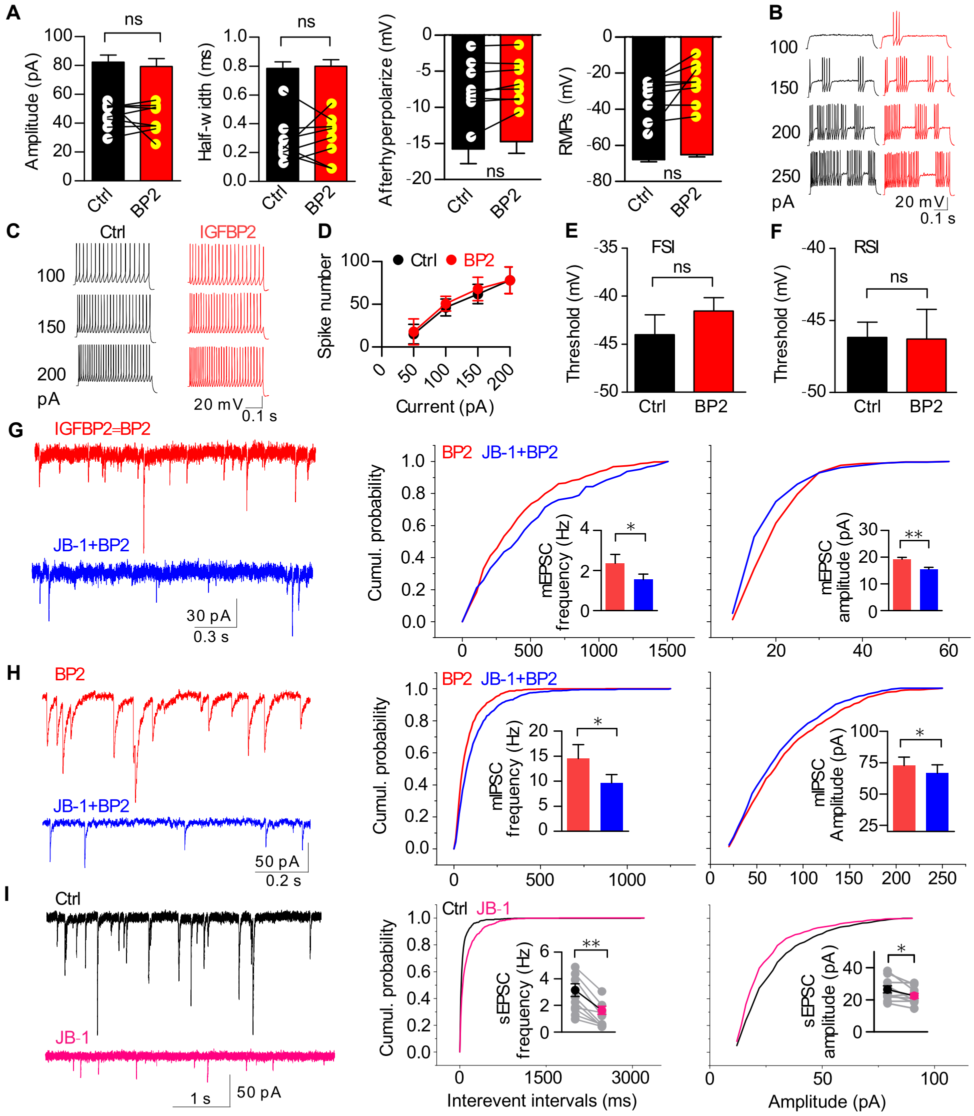


**Figure S2.** IGFR1 mediates the effect of IGFBP2. A) Effect of IGFBP2 on amplitude, half-width, after-hyperpolarization, and resting membrane potential (RMP) (n = 8). B) Exemplar CA1 RSI spikes. C,D) Exemplar CA1 FSI with spike number (n = 4). E,F) Voltage thresholds for FSIs (n = 5) and RSIs (n = 4). G-I) Representative traces with cumulative distributions of frequency and amplitude of mEPSCs/mIPSCs/sEPSCs in CA1 pyramidal neurons in response to an IGFR1 antagonist (JB-1, 1 µg/ml) (n = 8, 6, and 9). Paired two-tailed t-test except for panels G and H. *$P$ < 0.05, **$P$ < 0.01; data are mean ± SEM.

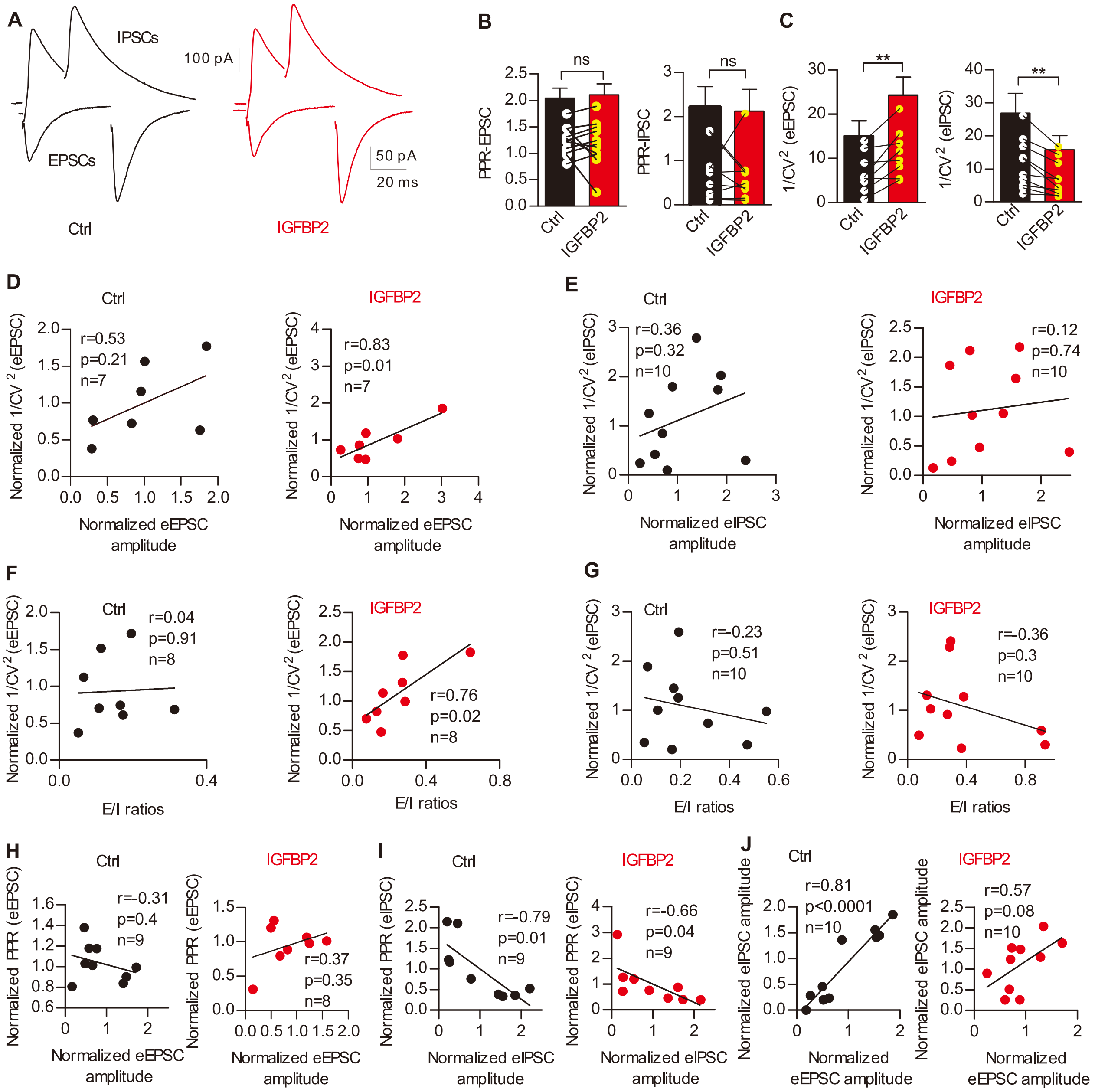


**Figure S3.** Increased numbers of functional synapses in response to IGFBP2. A) Exemplar eIPSCs and eEPSCs evoked by SC stimulation recorded at +10 mV and -60 mV, respectively. B) EPSC PPRs (n = 11) and IPSC PPRs (n = 10) were not affected by IGFBP2. C) IGFBP2 enhanced 1/CV2 of eEPSCs (n = 8) and decreased 1/CV2 of eIPSCs (n = 10). D) Plots of normalized 1/CV2-eEPSC amplitude against normalized eEPSC amplitude after IGFBP2 treatment. E) As in D but for eIPSCs. F,G) Plots of E/I ratios against normalized 1/CV2-eEPSC and 1/CV2-eIPSC amplitude. H) Plots of normalized eEPSC amplitude against normalized eEPSC PPR. I) As in H, but for eIPSCs. J) Normalized eEPSC amplitudes plotted

against normalized eIPSC amplitudes. The best-fit linear regression line is shown. ** *P* <0.01, paired two-tailed t-test; data are mean ± SEM.

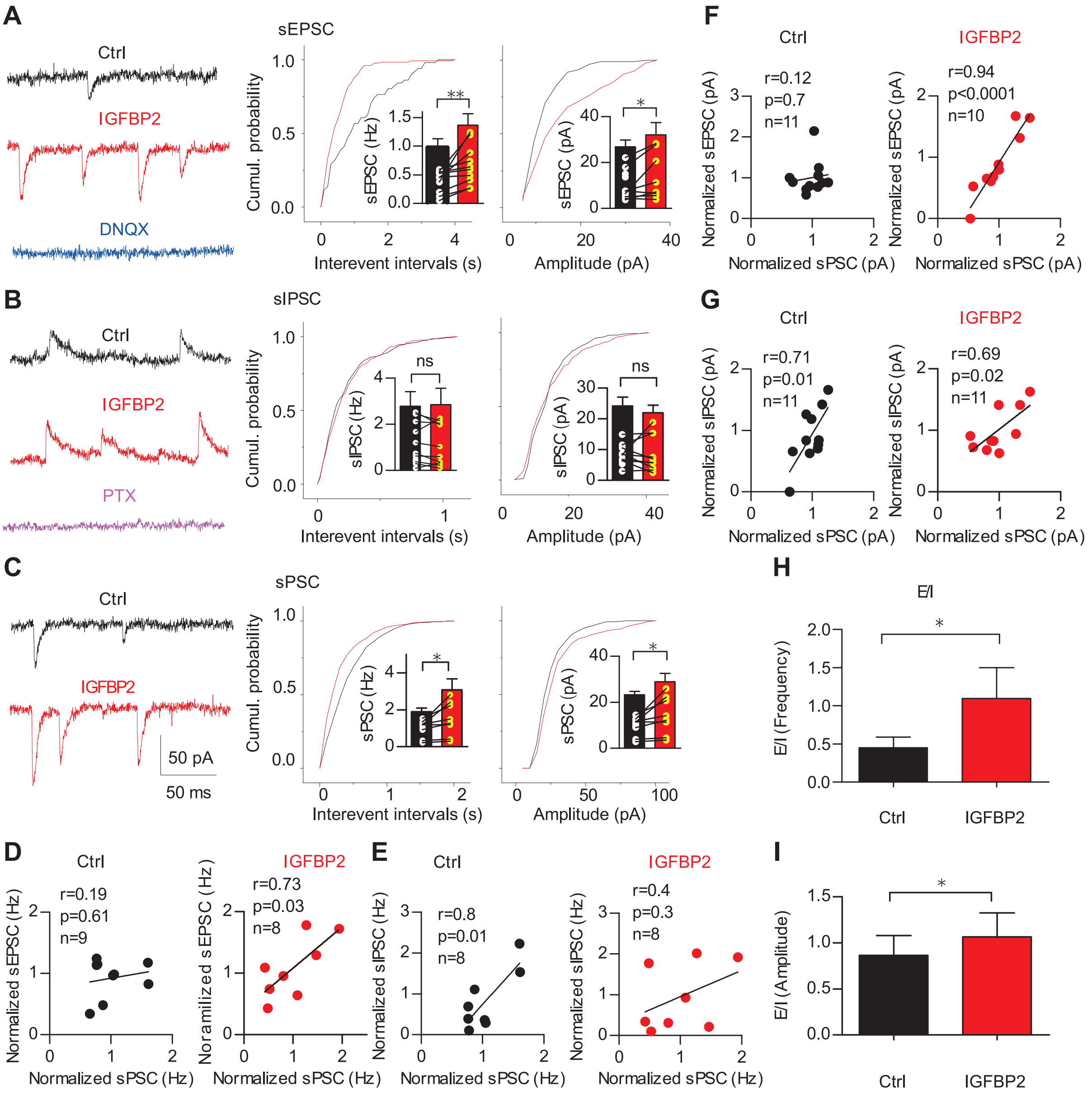


**Figure S4.** IGFBP2 increases EPSCs and not IPSCs. A-C) Exemplar sEPSCs/sIPSCs/sPSCs with cumulative distribution of frequency and amplitude after IGFBP2 treatment. D, E) Normalized sPSC frequency plotted against normalized-sEPSC and -sIPSC frequency before and after IGFBP2 treatment. F, G) As in D and E respectively, but for amplitudes. The best-fit linear regression line is shown. H, I) E/I ratios for frequency and amplitude. * *P* <0.05, ** *P* <0.01, paired two-tailed t-test; data are mean ± SEM.

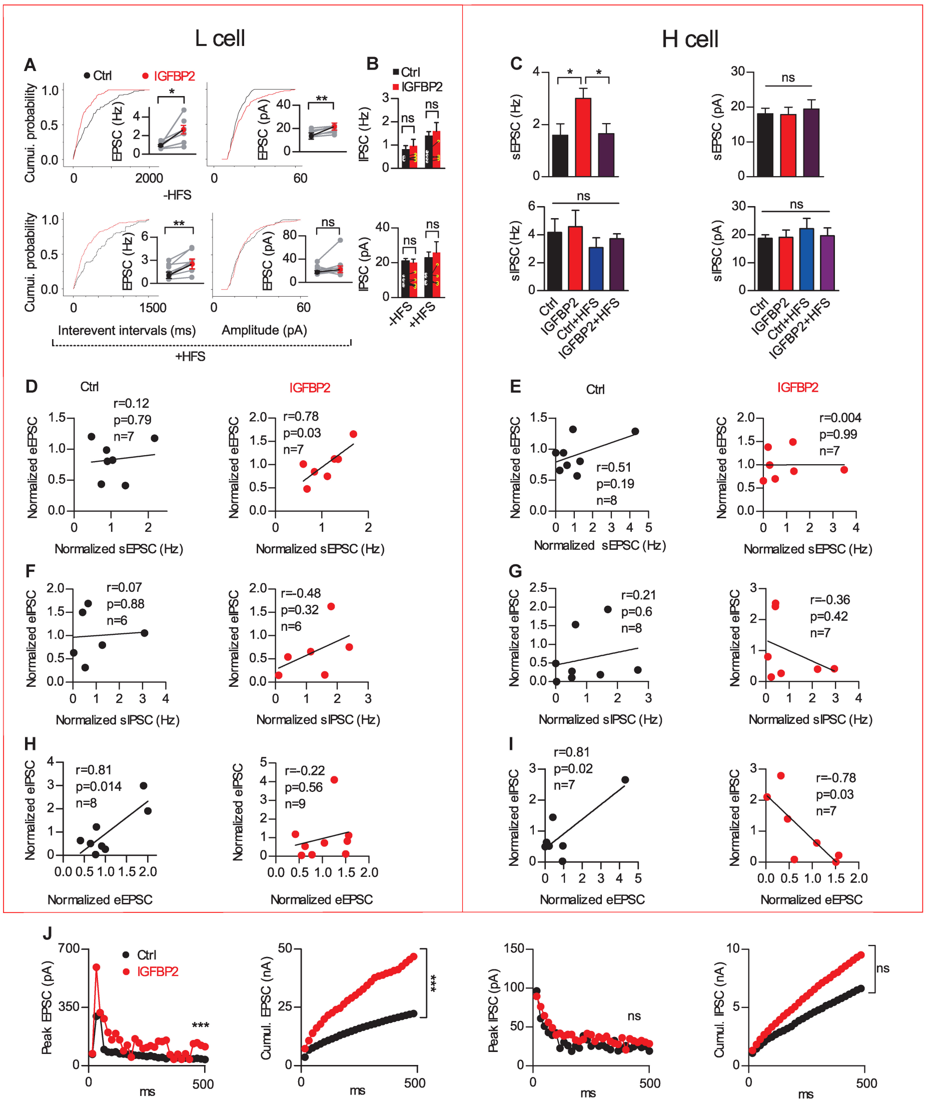


**Figure S5.** IGFBP2 induces plasticity in L cells for excitation but not inhibition. A) Cumulative distribution of sEPSC frequency (n = 8) and amplitude (n = 7) in L cells with or without HFS. B) As in A, value for sIPSCs (n = 5). C) sEPSC and sIPSC frequency and amplitude of H cells among the different groups (n = 6). D, E) Normalized sEPSC frequency plotted against normalized eEPSC amplitude either in L and H cells. F, G) As in D and E, respectively, but for IPSCs. H, I) Normalized eEPSC amplitude plotted against normalized eIPSC amplitude in L and H cells. The best-fit linear regression line is shown. J) Peak EPSC amplitudes, and cumulative EPSC amplitudes, peak IPSC amplitudes, cumulative IPSC amplitudes evoked by 60-Hz stimulation. *$P$ < 0.05, **$P$ < 0.01;***$P$ < 0.001, two-tailed t-test; data are mean ± SEM.

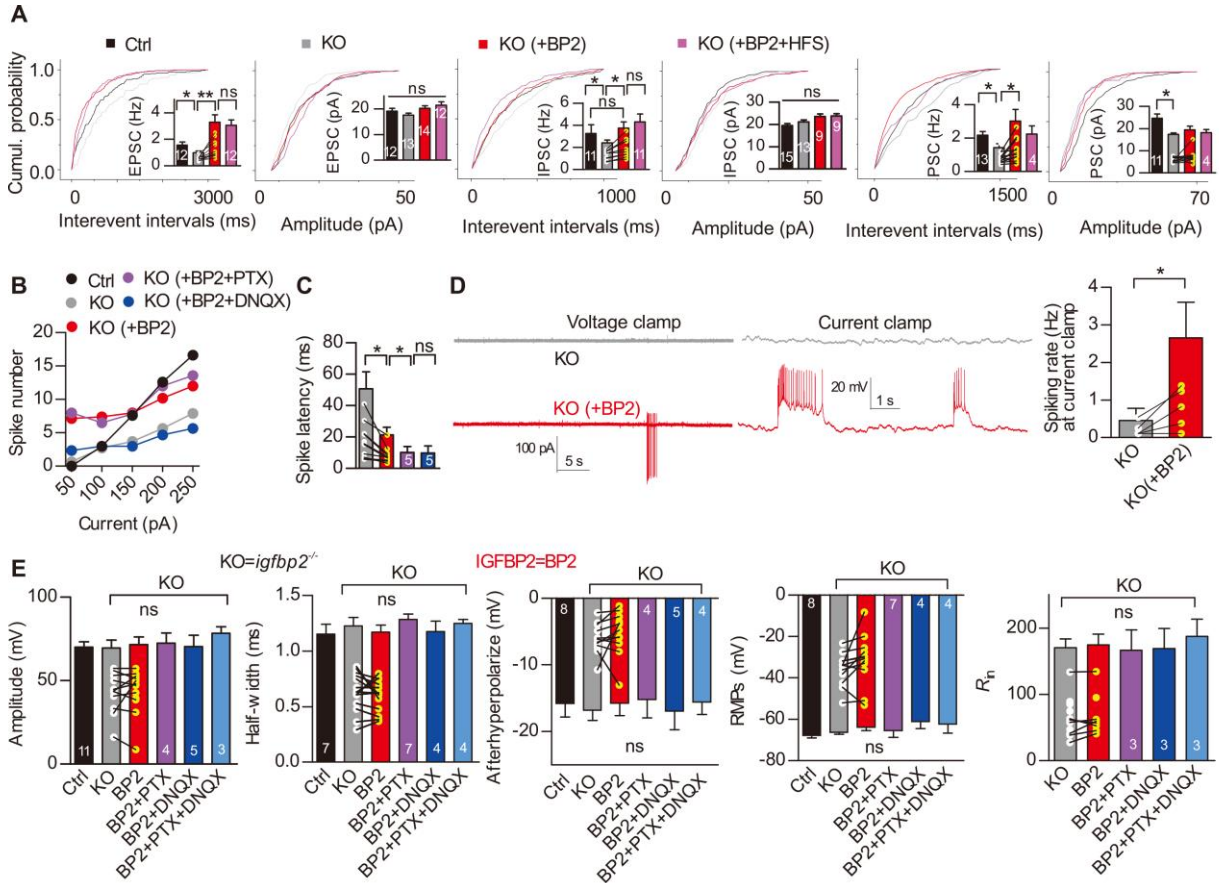


**Figure S6.** Properties of CA1 pyramidal neurons from *igfbp2*$^{-/-}$ mice. A) Cumulative distribution of sEPSC, sIPSC, and sPSC frequency and amplitude among groups. B) Amplitude, half-width, afterhyperpolarization, RMP, and input resistance ($R_{in}$) among the different groups (*igfbp2*$^{-/-}$, n = 8). C, D) Spike number and spike latency (n = 12) among groups. E) Exemplar cell-attached spontaneous spikes in CA1 pyramidal neurons in *igfbp2*$^{-/-}$ mice in voltage- and current-clamp modes. *$P$ < 0.05, **$P$ < 0.01, paired two-tailed t-test; data are mean ± SEM.

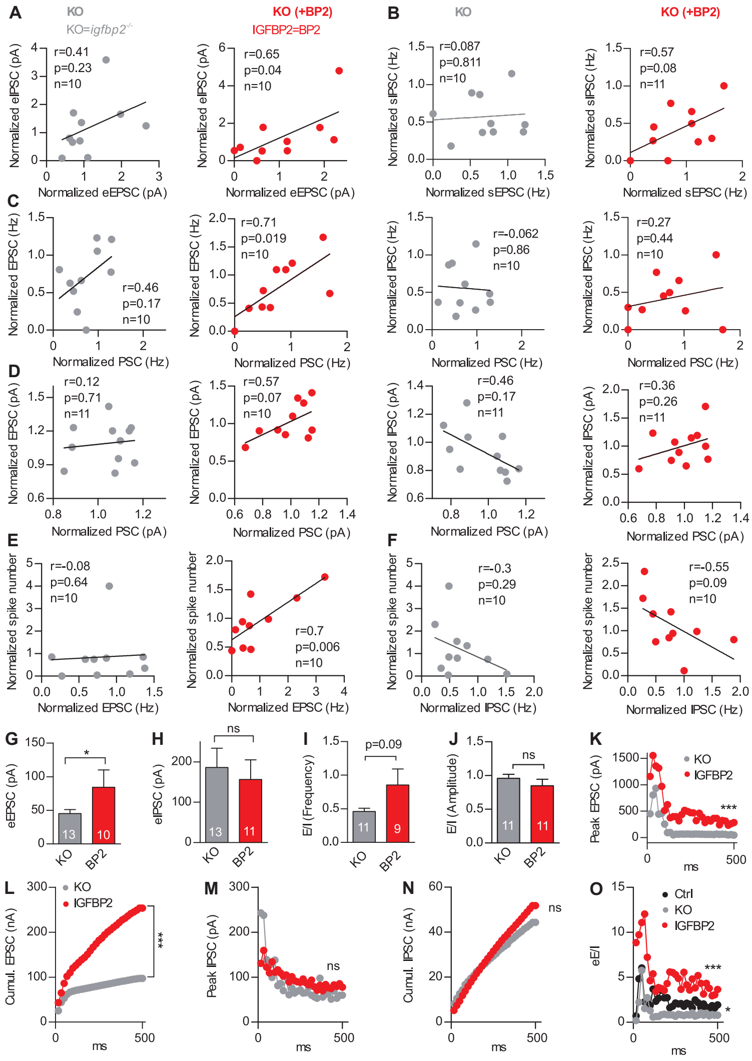


**Figure S7.** IGFBP2 rescued impaired E-I balance and E/I ratios. A) Normalized eEPSC amplitude plotted against normalized eIPSC amplitude from KO cells after IGFBP2 treatment. B) As in A, but for frequency. C) Normalized sPSC frequency plotted against normalized sEPSC and sIPSC frequency. D) As in C, but for amplitudes. E, F) Normalized sEPSC and sIPSC frequency plotted against normalized spike number. The best-fit linear regression line is shown. G, H) IGFBP2 treatment rescued eEPSCs but not eIPSCs from KO CA1 pyramidal neurons. I, J) Frequency but not amplitude E/I ratios were partially rescued after IGFBP2 treatment. K-O) Peak EPSC amplitudes, cumulative EPSC amplitudes, peak IPSC amplitudes, cumulative IPSC amplitudes, and E/I ratios in CA1 pyramidal neurons from *igfbp2*$^{-/-}$ mice (n = 18) evoked by 60-Hz stimulation. ***$P < 0.001$, the two-tailed t-test; data are mean ± SEM.

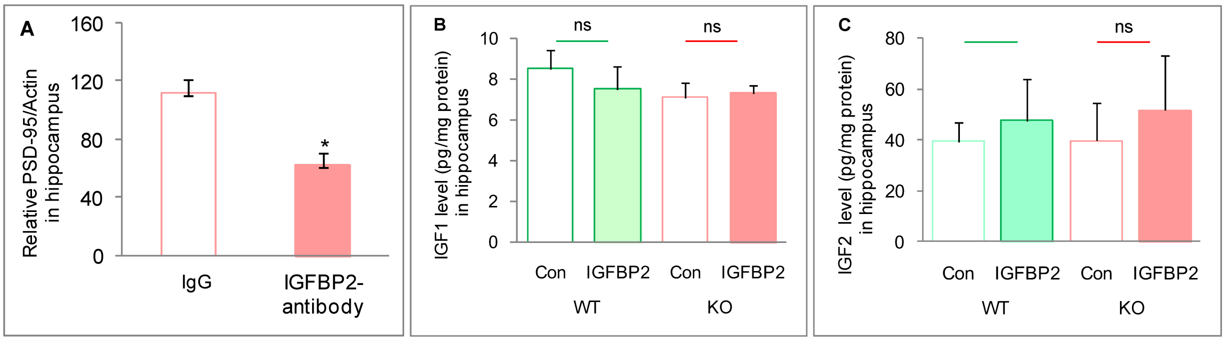


**Figure S8.** The hippocampal protein level was examined in mice after IGFBP2 antibody and IGFBP2 microinjection. A) PSD-95 decreased after microinjection of IGFBP2 antibody, n = 5-7, *$P$ < 0.05, data are mean ± SEM. B, C) No change is seen in IGF1 and IGF2 levels in IGFBP2 application in WT and KO mice, n = 5-6, two-tail t-test; data are mean ± SD.

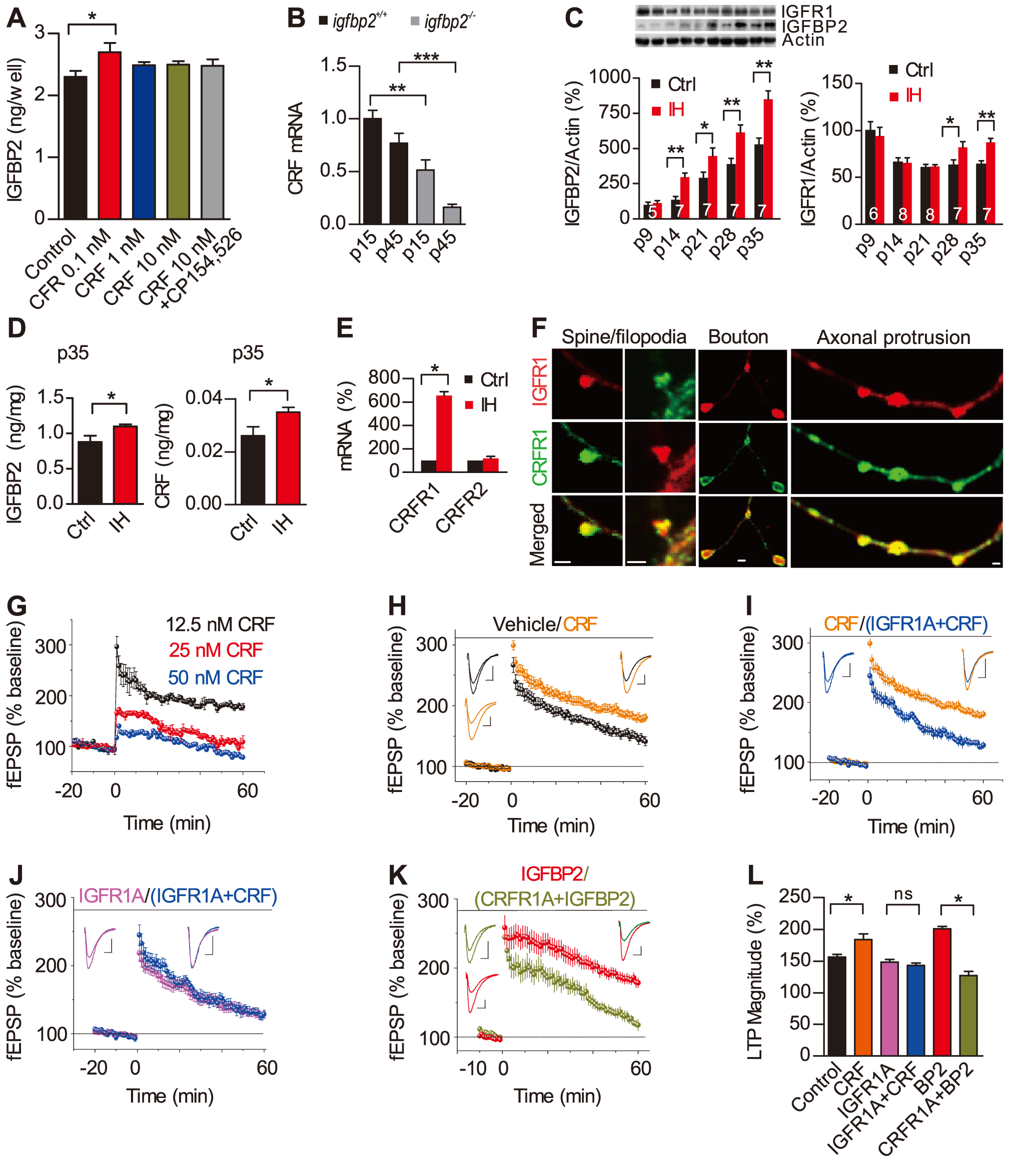

**Figure S9.** Interplay between IGFBP2 and CRF. A) CRF (0.1 nM) induced IGFBP2 release from cultured hippocampal neurons (n = 4, ANOVA and t-test). B) CRF mRNA expression in hippocampus from *igfbp2*$^{+/+}$ and *igfbp2*$^{-/-}$ mice. C, D, E) IGFBP2, IGFR1, CRF, and CRFR expression in p35 hippocampus during intermittent hypoxia (IH) (ANOVA). F) IGFR1 (red) co-localized with CRFR1 (green) on dendritic spines/filopodia and hippocampal boutons (scale bars, 1 µm). G-K) Changes in time-course of field EPSP slope in the presence of CRF (12.5 nM) (n = 11), IGFR1A + CRF (n = 10), IGFR1A (n = 10) and CRFR1A + IGFBP2 (n = 7) in hippocampal slices from p14 to p17 mice. L) Comparison of LTP amplitudes among different groups (ANOVA). $*P < 0.05$, $**P < 0.01$, $***P < 0.001$, the two-tailed t-test; data are mean ± SEM.

**Toc Figure**

IGFBP2 regulates plasticity in a cell-type-specific manner at excitatory synapses in the hippocampus, facilitates LTP by IGFR1 and NMDAR-s/eEPSCs, and rescues LTP and cognitive performance and excitation in KO mice.

**Experimental Section**

*Cell Identification*: Pyramidal neurons in the CA1 region were identified by the distinctive pyramidal shape of their soma with spiking behavior.[1] Fast-spiking interneurons (FSIs) were identified by electrophysiological properties (short action potential duration, higher frequency, shorter inter spike intervals (ISI) .**[2,3]** Regular -spiking interneurons (RSIs) were identified by electrophysiological properties (initial high-frequency spike output that declined to lower sustained frequencies with longer ISI than FSI). [3] (in Supp Material, Fig S2. E, F ).

*Extracellular Recording*: Field EPSPs were evoked at 0.05 Hz as previously described [4] and recorded with an aCSF-filled pipette (1-2 MΩ) positioned in the stratum radiatum of CA1; SC inputs were stimulated with monophasic pulses using a bipolar concentric electrode placed in CA3. LTP was induced by HFS consisting of four 1-s trains of 100-Hz pulses, delivered 20 s apart or four trains delivered 5 min apart, with the stimulus intensity set at 20-30% of the spike threshold. Field potentials were analyzed using Origin8 software. All reagents introduced during HFS were present for the remainder of the experiment. All plasticity experiments are presented as responses normalized to the average of the 20-min baseline. Every third trace (1-min intervals) is shown in graphs due to size limitations. LTP was quantified as the percentage change in the average slope of the field EPSP taken from 50 to 60 min. The two-tailed, unpaired Student's *t*-test or one-way ANOVA was used to determine significance. The field PPR was assessed by applying two pulses separated by inter-stimulus intervals of 50 ms, and was expressed as the increase in the leading slope of the second fEPSP

relative to the first. We recorded 15-30 individual traces before HFS or after 1 h of LTP recording in the presence or absence of IGFBP2 to measure average field PPRs.

*Morris Water Maze*: Morris water maze tests were performed using adult (p45/p60) male control (C57BL/6J) wild-type and *igfbp2*$^{-/-}$ mice. Mice were handled for 5 min each for 3 consecutive days before beginning experiments. The maze consisted of a large circular tank (1 m in diameter, 0.5 m high) of water at 25 ± 1°C made opaque by the addition of non-toxic water-based white paint. An escape platform (11 cm in diameter) was submerged 0.5-1 cm below the water surface in the center of one of the four quadrants and remained in this position for each mouse. Several visual cues were placed on the walls of the behavioral room as spatial references. An automated tracking system (DigBehv-MWM; Jiliang Software Technology Co. Ltd., Shanghai, China) monitored performance using the following parameters: escape latency (finding the submerged platform), swimming speed, and a visual sensitivity test. Mice were trained for 4 days with 3 trials per day separated by ~ 30 min prior to the first training trial; mice were given a single habituation trial without the platform to assess any spatial bias. Trials lasted 60 s and mice that did not find the platform within that time were guided to the platform by the experimenter. Once on the platform, mice were left for an additional 10 s before being removed. Start locations were pseudo-randomized so that each was used once per session and the sequence in any session was never used twice. A probe test was performed after the last trial on day 4. In this test the platform was removed and animals were allowed to swim freely for 30 s, during which the percentage of time spent in each quadrant and the swimming speed were recorded. Following the probe trial, visual and sensorimotor skills were assessed by 3 visible platform trials. The top of the platform was positioned 0.5-1 cm above the surface of the water and a green flag on a stand was attached to it to provide a strong visible cue. The position of the visible platform varied from trial to trial.[5]

**References**

[1] N. P. Staff, H. Y. Jung, T.Thiagarajan, M.Yao, N. Spruston, *J. Neurophysiol.* **2000**, *84*, 2398.

[2] H. Hu, J.Gan, P.Jonas, *Science* **2014**,*345*, 1255263.

[3] A. Gupta, Y. Wang, H. Markram, *Science* **2000**,*287*, 273.

[4] L. J. Volk, J. L. Bachman, R. Johnson, Y. Yu, R. L. Huganir, *Nature* **2013**, *493*, 420.

[5] X. J. Lu, X. Q. Chen, J. Weng, H. Y. Zhang, D. T. Pak, J. H. Luo, J. Z. Du, *Neuroscience* **2009**, *162*, 404.
